# Genome-Wide Network Analysis of Above- and Below-Ground Co-growth in *Populus euphratica*

**DOI:** 10.34133/plantphenomics.0131

**Published:** 2024-01-05

**Authors:** Kaiyan Lu, Huiying Gong, Dengcheng Yang, Meixia Ye, Qing Fang, Xiao-Yu Zhang, Rongling Wu

**Affiliations:** ^1^ College of Science, Beijing Forestry University, Beijing 100083, P. R. China.; ^2^ Yanqi Lake Beijing Institute of Mathematical Sciences and Applications, Beijing 101408, China.; ^3^ Center for Computational Biology, College of Biological Sciences and Technology, Beijing Forestry University, Beijing 100083, P. R. China.; ^4^ Faculty of Science, Yamagata University, Yamagata 990, Japan.

## Abstract

Tree growth is the consequence of developmental interactions between above- and below-ground compartments. However, a comprehensive view of the genetic architecture of growth as a cohesive whole is poorly understood. We propose a systems biology approach for mapping growth trajectories in genome-wide association studies viewing growth as a complex (phenotypic) system in which above- and below-ground components (or traits) interact with each other to mediate systems behavior. We further assume that trait–trait interactions are controlled by a genetic system composed of many different interactive genes and integrate the Lotka-Volterra predator–prey model to dissect phenotypic and genetic systems into pleiotropic and epistatic interaction components by which the detailed genetic mechanism of above- and below-ground co-growth can be charted. We apply the approach to analyze linkage mapping data of *Populus euphratica*, which is the only tree species that can grow in the desert, and characterize several loci that govern how above- and below-ground growth is cooperated or competed over development. We reconstruct multilayer and multiplex genetic interactome networks for the developmental trajectories of each trait and their developmental covariation. Many significant loci and epistatic effects detected can be annotated to candidate genes for growth and developmental processes. The results from our model may potentially be useful for marker-assisted selection and genetic editing in applied tree breeding programs. The model provides a general tool to characterize a complete picture of pleiotropic and epistatic genetic architecture in growth traits in forest trees and any other organisms.

## Infographic

**Figure:**
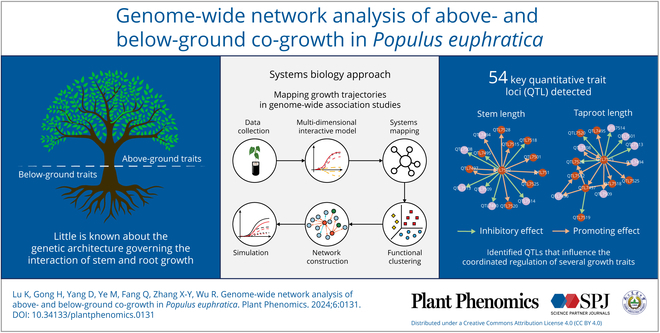

## Introduction

The growth of woody plants is a complex dynamic process involving coordination of resource allocation and signal transmission between above-ground stem and below-ground roots to adapt to changing environments [1–6]. Genes play a crucial role in shaping phenotypes during the biological process [7,8]. There has been much interest in explaining the intrinsic mechanism of plant growth and development from the perspective of gene regulation and gene interaction in recent years [9–13]. Some researchers have discerned that several genes drive the growth of phenotypic characteristics through biogenetics [14–16]. Some classical methods have emerged including transcriptome analysis, validation of the effect of genes employing transgenic means, genetic mapping, genome-wide association studies (GWASs), and so on [17–21]. Nevertheless, lots of current studies mainly focus on quantitative trait loci (QTLs) that regulate 1 or 2 traits, ignoring the integrity of organs [22,23]. On the other hand, a majority of methods pay more attention to the function of a few key genes, which can only explain a tiny fraction of phenotypic growth [24–26]. To date, we still know little about the overall genetic structure that governs the interaction and coordination of stem and root growth.

A deeper genetic understanding of complex traits requires considering the interactions among genes as a cohesive whole [27,28]. Boyle et al. [29] propose a whole gene model theory that traits or diseases are controlled by most genes carried by organisms. The genetic interaction network should not ignore the contribution of each gene, and every gene participates in life activities, although they are not as important as key genes. However, there still is a formidable challenge in establishing genetic networks on the whole genome [30–32]. With the advent of high-throughput genotyping technology, genotyping with thousands or even millions of single-nucleotide polymorphisms (SNPs) in each sample is a common phenomenon. Considering the interactions among complex traits and plant integrity on such a big dataset, how can we explore genes that regulate the formation of complex phenotypes and clarify the genetic mechanisms?

In this paper, we establish a computational model to excavate the genes that govern the process and pattern of development over time and infer multilayer large-scale interactive networks from genetic linkage. Our model integrates systems mapping, functional clustering [33], and evolutionary game theory [34,35] to figure out genetic contribution and topological structure underlying phenotypic formation. Considering the interaction among 4 above- and below-ground traits, i.e., stem length (ST), taproot length (TAP), lateral root length (LA), and average lateral root length (ALA), the multi-dimensional interactive model (MDIM) can describe how components interact with each other and show different growth characteristics. The major merit of MDIM lies in its ability to take into account the dynamic growth interaction of multiple traits and explore the genetic regulatory mechanisms behind it. We further assume that interactions among traits are controlled by a genetic system composed of many different interacting genes. Thus, we build a multilayer large-scale interactive network that covers all pairwise links of SNPs on the whole genome to chart the detailed genetic mechanism of above- and below-ground co-growth. Modularization theory [36,37] and functional clustering make it possible to operate on large-scale data, and different modules are linked and further divided into sub-modules and sub-submodules. Modularization has effectively reduced the dimensionality of large-scale genomic data and greatly reduced the computational difficulty of directly establishing regulatory networks on such massive data. At the same time, it extracts the commonalities of genes with similar genetic effect characteristics. Further subdivision of the modules indicates that while taking into account commonalities, the individual characteristics of each gene are not abandoned. Generalized nonlinear Lotka-Volterra equation based on genetic effects is a quantitative way to measure the strength of relationship among SNPs or modules, in which the net genetic effect of one SNP is composed of 2 parts: independent genetic effects caused by the intrinsic ability of one SNP and the epistatic influences of other SNPs on it [38,39]. The idea of the equation is that each SNP is not an isolated individual, and its genetic effects are reflected by the joint action of itself and other SNPs that affect it. Our multilayer interaction network provides a powerful computational tool in the mechanism analysis of high-dimensional genome-wide gene expression datasets and explains the genetic control of complex traits from an all-round perspective.

Taking *Populus euphratica* as an example, we apply our new framework to systematically dissect the genetic architecture of the woody plant, which is of great importance for stabilizing the ecological balance in desert river areas, as well as regulating oasis climate and forming fertile forest soil [40]. In the harsh environment of high temperatures or drought, it can grow well-developed below-ground roots and tough above-ground stem.

## Materials and Methods

### Experimental design

We used published experimental data to validate the utility of our model [14]. The mapping population is a full-sib F1 family, which came from a cross between 2 dioecious trees of *P. euphratica*. The 2 parents grew naturally in Korla, Xinjiang, China (male parent 0046 in 85°14′10″–86°24′21″ E and female parent Pe-1 in 41°10′48″–41°21′36″N). In spring 2014, the flowering branches were cut from these 2 trees, and cultivated in water for artificial hybridization. More than 4 months after pollination, the catkins gradually ripened, and then the harvested seeds were cultivated in glass tubes (40 mm in diameter and 400 mm in length) under a sterile culture condition. Finally, 345 progeny germinated and grew into seedlings. Four phenotypes, namely, ST (mm), TAP (mm), LA (cm), and ALA (mm), were measured repeatedly for each progeny since seed germination, which ranged from 6 to 23 days after being planted in glass tubes. Measurements were undertaken 12, 14, or 16 times. To be specific, the ST and TAP of some seedlings were recorded at 16 time points: 1, 3, 5, 7, 18, 20, 22, 24 26, 28, 31, 34, 38, 47, 54, and 62 days after seed germination, respectively. ST and TAP of some other seedlings were recorded at 12 time points: 1, 3, 5, 7, 10, 12, 15, 18, 22, 31 38, and 45 days after seed germination. LA and number were recorded at 14 time points: 1, 3, 5, 7, 9, 11, 13, 16, 19, 23, 28, 32, 39, and 47 days after germination.

The collected leaves of the F1 generation were quick-frozen in liquid nitrogen and then stored in an ultra-low temperature freezer at −80°C. Genomic DNA was extracted using the TIANGEN DNA extraction kit (Beijing). DNA concentration and quality were determined by agarose gel electrophoresis and a Nanodrop 2000 spectrophotometer (Thermo Fisher Scientific, Wilmington, DE, USA). Qualified DNA samples were sent to Majorbio (Shanghai) for high-throughput sequencing using RAD technology. The sequencing platform was Illumina HiSeq2500. Finally, 8,305 SNPs in 19 linkage groups (lg) were obtained. All SNP markers can be divided into 6,886 testcross markers and 1,419 intercross hybrid markers according to Mendelian genetic segregation rules, respectively. All procedures followed the method used by Xu et al. [41].

### Statistical analysis

#### Multi-dimensional interactive model

In the growth and evolution process of trees, proper coordination and competition of plants’ component traits reach a certain degree of dynamic equilibrium to acquire more resources and adapt to a specific environment. Growth trade-off among different organs is analogous to the constraints between predators and prey in an ecological system. To quantify the dynamic feature of complex traits, we propose an MDIM about above-ground ST and below-ground TAP, LA, and ALA of seedlings for *P. euphratica*. The growth process of most organisms involves multiple “S”-shaped phases according to the multiphasic growth model, and the seedling stage is a single phase [30,42,43]. MDIM contains 2 parts: the independent growth and interactive growth of traits. The independent growth part is determined by its intrinsic characteristics, and it follows the characteristics of the logistic curve [44]:

$$\frac{\mathrm{dN}}{\mathrm{dt}}=r\cdot N\cdot \left(1-\frac{N}{K}\right)$$ (1)

where *N* represents the population quantity, *K* represents the maximum possible population size, and *r* represents the growth velocity.

The interactive part quantifies the allometric relationship among different components of stem and roots, and it derives from the Lotka–Volterra differential equation model, which was originally proposed to describe the characteristics of competition and cooperation of 2 species [24]:

$$\left\{\begin{array}{c} \frac{{\mathrm{dN}}_{1}}{\mathrm{dt}}=r_{1}\cdot N_{1}\cdot \left(1-\frac{N_{1}}{K_{1}}\right)-r_{1}\cdot \frac{N_{1}}{K_{1}}\cdot \alpha \cdot N_{2}\\ \frac{{\mathrm{dN}}_{2}}{\mathrm{dt}}=r_{2}\cdot N_{2}\cdot \left(1-\frac{N_{2}}{K_{2}}\right)-r_{2}\cdot \frac{N_{2}}{K_{2}}\cdot \beta \cdot N_{1} \end{array}\right.$$ (2)

where *N*_1_ and *N*_2_ represent the population quantity of 2 species, *K*_1_ and *K*_2_ represent the intrinsic-carrying capacity of 2 species, *r*_1_ and *r*_2_ represent the growth velocity of 2 species, *α* and *β* represent how 2 species affect each other.

By denoting trait 1, trait 2, trait 3, and trait 4 as 4 above- and below-ground traits, respectively, the MDIM is expressed as:

$$\left\{\begin{array}{c} \frac{{\mathrm{dN}}_{1}}{\mathrm{dt}}=\frac{{{\mathrm{dN}}_{1}}^{I}}{\mathrm{dt}}+\frac{{{\mathrm{dN}}_{1}}^{D}}{\mathrm{dt}}=r_{1}\cdot N_{1}\cdot \left(1-\frac{N_{1}}{K_{1}}\right)+\sum_{i=1,i\neq 1}^{4}\alpha_{1\leftarrow i}{N_{i}}^{s_{1\leftarrow i}}\\ \begin{array}{c} \frac{{\mathrm{dN}}_{2}}{\mathrm{dt}}=\frac{{{\mathrm{dN}}_{2}}^{I}}{\mathrm{dt}}+\frac{{{\mathrm{dN}}_{2}}^{D}}{\mathrm{dt}}=r_{2}\cdot N_{2}\cdot \left(1-\frac{N_{2}}{K_{2}}\right)+\sum_{i=1,i\neq 2}^{4}\alpha_{2\leftarrow i}{N_{i}}^{s_{2\leftarrow i}}\\ \frac{{\mathrm{dN}}_{3}}{\mathrm{dt}}=\frac{{{\mathrm{dN}}_{3}}^{I}}{\mathrm{dt}}+\frac{{{\mathrm{dN}}_{3}}^{D}}{\mathrm{dt}}=r_{3}\cdot N_{3}\cdot \left(1-\frac{N_{3}}{K_{3}}\right)+\sum_{i=1,i\neq 3}^{4}\alpha_{3\leftarrow i}{N_{i}}^{s_{3\leftarrow i}}\\ \frac{{\mathrm{dN}}_{4}}{\mathrm{dt}}=\frac{{{\mathrm{dN}}_{4}}^{I}}{\mathrm{dt}}+\frac{{{\mathrm{dN}}_{4}}^{D}}{\mathrm{dt}}=r_{4}\cdot N_{4}\cdot \left(1-\frac{N_{4}}{K_{4}}\right)+\sum_{i=1,i\neq 4}^{4}\alpha_{4\leftarrow i}{N_{i}}^{s_{4\leftarrow i}} \end{array} \end{array}\right.$$ (3)

where (*N*_1_, *N*_2_, *N*_3_, *N*_4_) represents the growth phenotype values of 4 traits. The independent growth part of each trait $\frac{{\mathrm{dN}}_{l}^{I}}{\mathrm{dt}}=r_{l}\cdot N_{l}\cdot \left(1-\frac{N_{l}}{K_{l}}\right)\left(l=1,2,3,4\right)$ is described by the maximum growth value (*K*_1_, *K*_2_, *K*_3_, *K*_4_), and the velocities of a trait (*r*_1_, *r*_2_, *r*_3_, *r*_4_) to grow to its maximum value (*r*_1_, *r*_2_, *r*_3_, *r*_4_). The interactive part of each trait is determined by the effect of the other 3 phenotypes, expressed as $\frac{{{\mathrm{dN}}_{l}}^{D}}{\mathrm{dt}}=\sum_{i=1,i\neq l}^{l}\alpha_{l\leftarrow i}{N_{i}}^{s_{l\leftarrow i}}\left(l=1,2,3,4\right)$; the degree of this interaction is described by interaction parameters *α*_*l*←*i*_ and scale parameters *s*_*l*←*i*_.

#### Statistical model of systems mapping

Systems mapping is an approach regarding complex phenotypes as a dynamic system that develops from functional mapping [45–47]. The constituent components interact in a way that can be quantitatively described by differential equations. Existing research is mainly limited to bivariate or even univariate, and the complexity of multiple traits is frequently neglected. To illustrate the process of how above-ground and below-ground traits interact, we design a full-sib mapping population (*P. euphratica*) of *n* trees. All samples are genotyped for *p* SNPs throughout the entire genome, and phenotyped for trait 1 (above-ground ST), trait 2 (below-ground TAP), trait 3 (below-ground LA), and trait 4 (below-ground ALA) at a series of time points.

Let a series of T-dimensional vectors *y*_*i*1_ = (*y*_*i*1_(1), *y*_*i*1_(2), …, *y*_*i*1_(*T*))*^T^*, *y*_*i*2_ = (*y*_*i*2_(1), *y*_*i*2_(2), …, *y*_*i*2_(*T*))*^T^*, *y*_*i*3_ = (*y*_*i*3_(1), *y*_*i*3_(2), …, *y*_*i*3_(*T*))*^T^*, and *y*_*i*4_ = (*y*_*i*4_(1), *y*_*i*4_(2), …, *y*_*i*4_(*T*))*^T^* represent the phenotypic values of individual *i* for the 4 traits at time *t* (*t* = 1, …, T), respectively. The likelihood function of *n* samples is expressed as

$$L_{0}\left(\vec{y},\;\right)=\prod_{i=1}^{n}f\left({\vec{y}}_{i}\right)=\prod_{i=1}^{n}f\left(y_{i1},y_{i2,}y_{i3},y_{i4}\right)$$ (4)

where ${\vec{y}}_{i}={\left({y_{i1}}^{T},{y_{i2}}^{T},{y_{i3}}^{T},{y_{i4}}^{T}\right)}^{T}$ is a 4T-dimensional vector, and $f\left({\vec{y}}_{i}\right)$ is the joint probability density function of 4 traits. Considering different alleles at a given QTL, we formulate the likelihood function of the phenotypic values as follows:

$$\begin{array}{c} \begin{array}{l} L_{1}\left(\vec{y}\right)=\prod_{j=1}^{J}\prod_{i=1}^{n_{j}}f_{j}\left({\vec{y}}_{1}\right)=\prod_{j=1}^{J}\prod_{i=1}^{n_{j}}f_{j}\left(y_{i1},y_{i2},y_{i3},y_{i4}\right)\\ =\prod_{j=1}^{J}\prod_{i=1}^{n_{j}}\frac{1}{{\left(2\pi \right)}^{\frac{1}{2}}{\left|\sum \right|}^{\frac{1}{2}}}\exp\left[-\frac{1}{2}{\left({\vec{y}}_{i}-{\vec{u}}_{j}\right)}^{T}\sum^{-1}\left({\vec{y}}_{i}-{\vec{u}}_{j}\right)\right] \end{array} \end{array}$$ (5)

where $f_{j}\left({\vec{y}}_{1}\right)$ is a 4T-dimensional multivariate normal distribution with mean vector ${\vec{u}}_{j}$ and covariance matrix Σ of individual *i* for allele *j* (*j* = 1, ..., *J*). The QTL carries *J* alleles, and the number of individuals for allele *j* is *n_j_*. The 4T-dimensional mean vector of allele *j* is

$$\begin{array}{c} \begin{array}{c} {\vec{\mu }}_{j}={\left({\vec{\mu }}_{j1},{\vec{\mu }}_{j2},{\vec{\mu }}_{j3},{\vec{\mu }}_{j4}\right)}^{T}\\ \;={\left(\mu_{j1}\left(1\right),\ldots ,\mu_{j1}\left(T\right);\mu_{j2}\left(1\right),\ldots ,\mu_{j2}\left(T\right);\mu_{j3}\left(1\right),\ldots ,\mu_{j3}\left(T\right);\mu_{j4}\left(1\right),\ldots ,\mu_{j4}\left(T\right)\right)}^{T} \end{array} \end{array}$$ (6)

and the 4*T* × 4*T* covariance matrix Σ is:

$$\sum =\left(\begin{array}{cccc} \sum_{11} & \sum_{12} & \sum_{13} & \sum_{14}\\ \sum_{21} & \sum_{22} & \sum_{23} & \sum_{24}\\ \sum_{31} & \sum_{32} & \sum_{33} & \sum_{34}\\ \sum_{41} & \sum_{42} & \sum_{43} & \sum_{44} \end{array}\right)$$ (7)

where $\sum_{k_{1}k_{2}}$ (*k*_1_ or *k*_2_ = 1, 2, 3, 4) on the diagonal is a *T* × *T* variance matrix of each trait, Σ_*k*_1_*k*_2__(*k*_1_ or *k*_2_ = 1, 2, 3, 4), and *k*_1_ ≠ *k*_2_ represents the covariance matrix between 2 traits, which is symmetric, i.e., $\sum_{k_{1}k_{2}}$ = $\sum_{k_{1}k_{2}}$. Here, the first-order structured antedependence (SAD (1)) model [48–50] is used to calculate the covariance matrix Σ, which is more efficient and flexible. The element $\sum_{k_{1}k_{2}}$(*k*_1_ or *k*_2_ = 1, 2, 3, 4) in Σ has the following structure:

$$\begin{array}{c} \sum_{k1k2}=\left(\begin{array}{cccc} \sigma_{k_{1}k_{2}}^{2}\left(1,1\right) & \sigma_{k_{1}k_{2}}\left(1,2\right) & \cdots & \sigma_{k_{1}k_{2}}\left(1,T\right)\\ \sigma_{k_{1}k_{2}}\left(2,1\right) & \sigma_{k_{1}k_{2}}^{2}\left(2,2\right) & \cdots & \sigma_{k_{1}k_{2}}\left(2,T\right)\\ \vdots & \vdots & \ddots & \vdots \\ \sigma_{k_{1}k_{2}}\left(T,1\right) & \sigma_{k_{1}k_{2}}\left(T,2\right) & \cdots & \sigma_{k_{1}k_{2}}^{2}\left(T,T\right) \end{array}\right) \end{array}$$ (8)

when *k*_1_ = *k*_2_, $\sigma_{k_{1}k_{2}}^{2}\left(t,t\right)=\sigma_{k}^{2}\left(t,t\right)$, and $\sigma_{k_{1}k_{2}}^{2}\left(t_{1},t_{2}\right)=\sigma_{k}^{2}\left(t_{1},t_{2}\right)$, all elements can be obtained by the following equations:

$$\left\{\begin{array}{c} \begin{array}{c} \sigma_{k}^{2}\left(t,t\right)=\gamma_{k}^{2}\frac{1-\phi_{k}^{2t}}{1-\phi_{k}^{2}}\\ \sigma_{k}\left(t_{1},t_{2}\right)=\gamma_{k}^{2}\phi_{k}^{t_{2}-t_{1}}\frac{1-\phi_{k}^{2t_{1}}}{1-\phi_{k}^{2}}\;,t_{2}>t_{1} \end{array}\\ \begin{array}{c} \sigma_{k_{1}k_{2}}^{2}\left(t,t\right)=\gamma_{k_{1}}\gamma_{k_{2}}\rho_{k_{1}k_{2}}\\ \sigma_{k_{1}k_{2}}\left(t_{1},t_{2}\right)=\gamma_{k_{1}}\gamma_{k_{2}}\rho_{k_{1}k_{2}}\frac{\phi_{k_{2}}^{t_{2}-t_{1}}-\phi_{k_{1}}^{t_{1}}\phi_{k_{2}}^{t_{2}}}{1-\phi_{k_{1}}\phi_{k_{2}}} \end{array} \end{array}\right.$$ (9)

where $\gamma_{k}^{2}$ is the innovation variance of trait *k*, antedependence parameters *ϕ_k_* are determined by the characteristics of the trait themselves, and $\rho_{k_{1}k_{2}}$ represents the correlation between trait *k*_1_ and trait *k*_2_. All parameters used to calculate the covariance matrix Σ are denoted as parameter set *τ*. The mean vector ${\vec{u}}_{\;j}$ can be estimated through a parameter set θ*_j_* = (*r_j_*, *K_j_*, *α*_*l*_*j*←*i_j_*__), (*i_j_*, *l_j_* = 1, 2, 3, 4 and *i_j_* ≠ *l_j_*) in MDIM; specifically, after obtaining the best parameter estimation of MDIM, the solution for $\left({\vec{N}}_{1},{\vec{N}}_{2},{\vec{N}}_{3},{\vec{N}}_{4}\right)$ is the mean vector. We implement an iterative optimization strategy based on maximum likelihood estimation by using the iterative ideas in the EM (Expectation–Maximization) algorithm for reference [51,52] (see Supplementary Materials for details). The iterative optimization strategy obtains the maximum likelihood estimates (MLEs) with regard to the parameter set Θ*_j_* = (θ*_j_*, *τ*) and the fourth-order Runge–Kutta algorithm solves the differential equations in MDIM during the optimization process.

#### Identification of key QTLs

We test the statistical significance of an SNP by a hypothesis, that is, whether an SNP regulates the growth of above-ground and below-ground and their interaction statistically:

$$H_{0}:\Theta_{j}\equiv \Theta ,\forall j=1,\cdots ,J\;\text{versus}\;H_{1}:\Theta_{j}\neq \Theta ,\exists j=1,\cdots ,J$$ (10)

where the null hypothesis indicates that the parameters of the growth equation are independent of alleles, and the alternative hypothesis means that the parameter sets are distinguishing for different alleles. A hypothesis test is performed for each SNP. Specifically, for an SNP with *J* alleles, we can calculate the likelihood values *L*_0_ of *H*_0_ (Eq. 4) under Θ and *L*_1_ of *H*_1_ (Eq. 5) under Θ*_j_* (*j* = 1,..., *J*), and statistic *LR*, namely, the likelihood ratio value:

$$\mathrm{LR}=-\;2\text{log}\left(L_{0}/L_{1}\right)$$ (11)

*LR* approximately obeys the *χ*^2^ distribution when the null hypothesis is correct. The degree of freedom of the *χ*^2^ distribution is the difference in the number of parameters between the null hypothesis *H*_0_ and the alternative hypothesis *H*_1_. Let *N*_Θ_ represent the number of parameters under the null hypothesis, then the number of parameters under the alternative hypothesis is *J*^∗^*N*_Θ_, and the degree of freedom of the *χ*^2^ distribution is (*J* − 1) ∗ *N*_Θ_. The *p*-value can be calculated based on the distribution of *LR* and determine if it is greater than the critical thresholds. We use the false discovery rate (FDR) to adjust the critical value, control the false positive rate, and ensure more accurate results.

#### Multivariate functional clustering

We calculate genetic standard deviations of SNPs, i.e., genetic effects to describe the genetic impact on the development of phenotypes. The genetic standard deviation of an SNP at time point *t* is expressed as:

$$g\left(t\right)=\sqrt{\frac{\sum_{j=1}^{J}{\left(\frac{1}{n_{j}}\sum_{i=1}^{n_{j}}{y_{1}}^{j}\left(t\right)-\frac{1}{n_{j}}\sum_{j=1}^{J}\frac{1}{n_{j}}\sum_{i=n_{1}}^{n_{j}}{y_{1}}^{j}\left(t\right)\right)}^{2}}{J}}$$ (12)

where *y*_1_*^j^*(*t*) represents the phenotypic values of individual *i* at time *t* with allele *j*. The meaning of *J* and *n_j_* are the same as those of Eq. 5. To identify different dynamic patterns of gene expression from a large number of SNPs, functional clustering is introduced to divide *p* SNPs into *M* modules with similar genetic effects. This clustering allows us to construct smaller interconnected networks from one large network. In this paper, functional clustering is extended to multivariate. The genetic effects of SNP *k* (*k* = 1, …, *p*) of 4 traits are expressed as:

$$\begin{array}{l} g_{1k}={\left(g_{1k}\left(1\right),\cdots ,g_{1k}\left(T\right)\right)}^{T},g_{2k}={\left(g_{2k}\left(1\right),\cdots ,g_{2k}\left(T\right)\right)}^{T},\\ g_{3k}={\left(g_{3k}\left(1\right),\cdots ,g_{3k}\left(T\right)\right)}^{T},g_{4k}={\left(g_{4k}\left(1\right),\cdots ,g_{4k}\left(T\right)\right)}^{T}. \end{array}$$ (13)

where *g*_1*k*_, *g*_2*k*_, *g*_3*k*_, and *g*_4*k*_ are T-dimensional vectors. The likelihood is formulated as:

$$L_{2}\left(g_{1},g_{2},g_{3},g_{4}\right)=\prod_{k=1}^{p}\sum_{m=1}^{M}\left[\omega_{m}f_{m}\left(g_{1k},g_{2k},g_{3k},g_{4k};\Phi_{m}\right)\right]$$ (14)

where *ω_m_* is the prior probability representing the proportion of module *m*, and satisfies $\sum_{m=1}^{M}\omega_{m}=1$. *f_m_*(*g*_1*k*_, *g*_2*k*_, *g*_3*k*_, *g*_4*k*_; Φ*_m_*) is a multivariate normal distribution, and Φ*_m_* is a parameter set that can be estimated by the iterative optimization strategy based on maximum likelihood estimation, which constructs the 4T-dimensional mean vector of the corresponding module:

$$\begin{array}{c} u_{m}=(u_{m1}\left(1\right),\cdots u_{m1}\left(T\right);u_{m2}\left(1\right),\cdots ,\\ \;u_{m2}\left(T\right);u_{m3}\left(1\right),\cdots ,u_{m3}\left(T\right);u_{m4}\left(1\right),\cdots ,u_{m4}\left(T\right){)}^{T} \end{array}$$ (15)

and Σ*_g_* is a 4*T* × 4*T* covariance matrix.

The mean vector and covariance matrix are solved using the Legendre Orthogonal Polynomial (LOP) equation and the SAD (1) model, respectively. At each iteration, the posterior probability of SNP *k*, which belongs to module *m*, can be calculated by the following formula:

$$\Omega_{m\left|k\right.}=\frac{\omega_{m}f_{m}\left(g_{1k},g_{2k},g_{3k},g_{4k};\Phi_{m}\right)}{\sum_{m^{\prime }}^{M}\omega_{m^{\prime }}f_{m^{\prime }}\left(g_{1k},g_{2k},g_{3k},g_{4k};\Phi_{m^{\prime }}\right)}$$ (16)

Weight coefficient *ω_m_*, i.e., the proportion of module *m*, is:

$$\omega_{m}=\frac{\sum_{k=1}^{p}\Omega_{m\left|k\right.}}{p}$$ (17)

The optimal number of modules is determined by penalized likelihood criteria such as AIC (Akaike information criterion) or BIC (Bayesian information criterion).

#### Multilayer network construction

Genome-wide networks help us to have a more comprehensive understanding of the genetic mechanisms behind phenotypic variation. The crucial steps in constructing genetic networks are smoothing data, selecting variables, and solving linear differential equations. LOP smooths the genetic effect first. Select the appropriate order number of LOP, and then polynomial functions that were composed of the base of orthogonal polynomials can approach target functions using the least square method to achieve the purpose of smoothing. Network sparsity theory suggests that the number of modules required to maintain the stability of the network structure is limited, which can be filtered by variable selection in the regression model:

$$g_{\cdot m}\left(t\right)=a_{m}+\sum_{m^{\prime }=1,m^{\prime }\neq m}^{M}b_{m^{\prime }}g_{\cdot m^{\prime }}\left(t\right)+e_{m}\left(t\right)$$ (18)

where *a_m_* is a constant, $b_{m^{\prime }}$ is the regression coefficient of the variable $m^{\prime },g_{\cdot m}\left(t\right)\left(g_{\cdot m}\left(t\right)=g_{1m}\left(t\right),g_{2m}\left(t\right),g_{3m}\left(t\right),g_{4m}\left(t\right)\right)$ represents the genetic effect of module *m* for the target trait, $\begin{array}{c} g_{\cdot m}\left(t\right)\left(g_{{\cdot m}^{\prime }}\left(t\right)=g_{{1m}^{\prime }}\left(t\right),g_{{2m}^{\prime }}\left(t\right),g_{{3m}^{\prime }}\left(t\right)\;\text{and}\;g_{{4m}^{\prime }}\left(t\right)\right) \end{array}$ represents the genetic effect of module $m^{\prime }$, and *e_m_*(*t*) represents residual. The genetic effect of module *m* is the average of the genetic effects of all SNPs contained in module *m*. LASSO-based variable selection is implemented to confirm the set of modules that have the greatest impact on the target module. The determination of penalty parameters and the solution of the lasso problem are implemented by the R package *grplasso*.

**Fig. 1.**
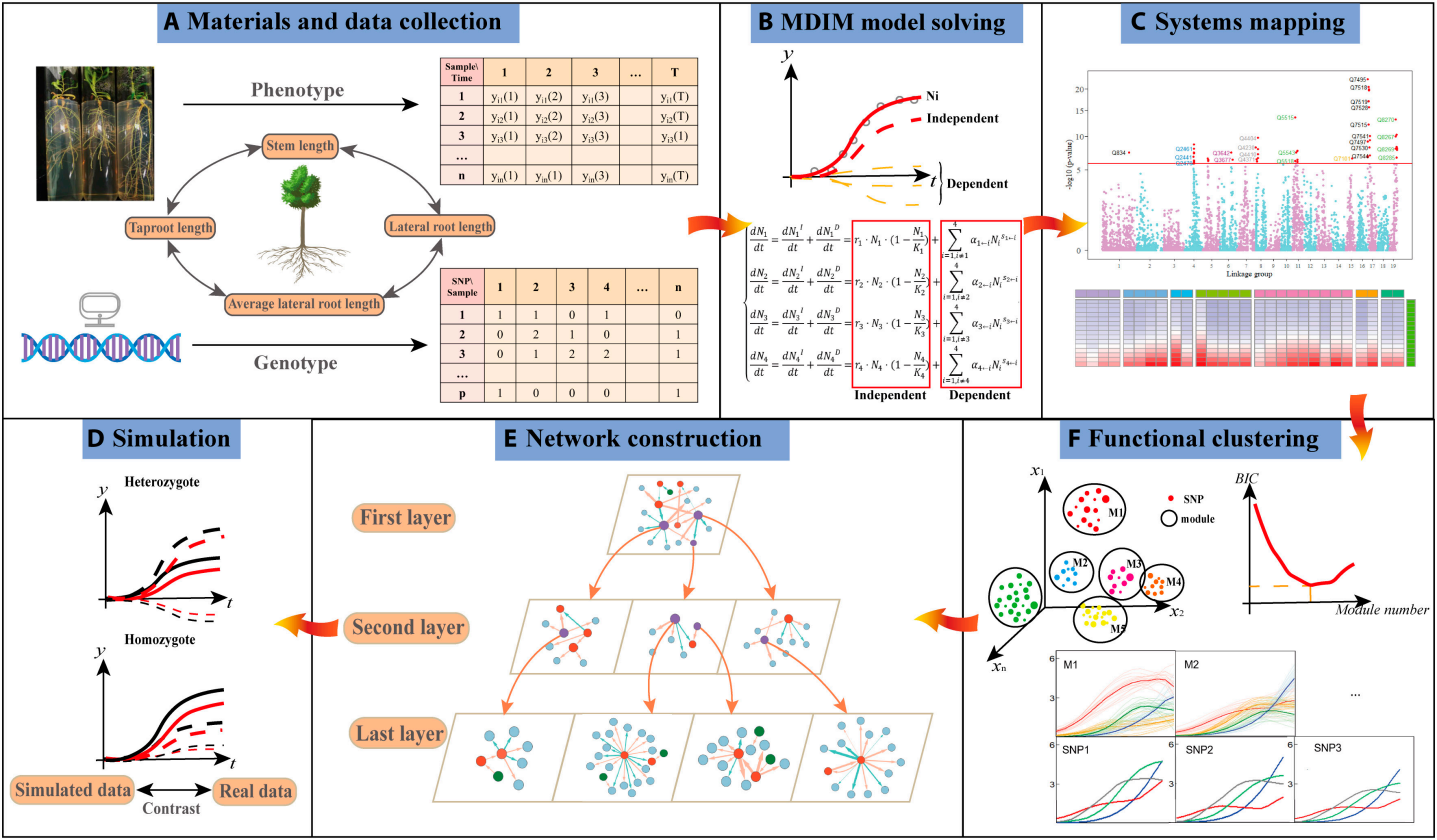
The workflow of genome-wide multilayer networks that mediate complex dynamic traits. (A) Collection of time-series phenotypic data and alleles of stem height, taproot length, lateral root length, and average lateral root length in *Populus euphratica*. (B) Construct and fit the multi-dimensional interactive model (MDIM). (C) Multivariate systems mapping to identify key QTLs regulating the growth of multiple traits and their interactions. (D) Obtain the optimal number of modules for functional clustering according to the BIC criterion. (E) Build a network (bidirectional, signed, and weighted) among modules (nodes) and subdivide the modules further until a node represents an SNP. (F) Simulation experiments to verify the validity of our model.

Utilizing the replication factor equation, dynamic evolutionary game theory explains interactions among loci [34] and describes how a player behaves differently based on changes in its strategy and other players’ strategies over time to simulate the evolution in a population [53,54]. The replication factor equation treats the performance of the explanatory variable as the sum of its strategy and its interacting opponent’s strategy [35]. Here, we propose a nonlinear differential equation system to describe independent and dependent parts:

$$\frac{{\mathrm{dg}}_{\cdot m}}{\mathrm{dt}}=G_{m}^{0}\left(g_{\cdot m};\varphi_{m}\right)+\sum_{m^{\prime }=1,m^{\prime }\neq m}^{M}G_{m\leftarrow m^{\prime }}\left(g_{\cdot m^{\prime }};\varphi_{m\leftarrow m^{\prime }}\right),m=1,\cdots ,M$$ (19)

where *g*_⋅*m*_ represents the net genetic effect of module *m* on one trait, which is decomposed into 2 parts: $G_{m}^{0}\left(g_{\cdot m};\varphi_{m}\right)$ as a function of time, describing the independent parts of genetic effect; $\sum_{m^{\prime }=1,m^{\prime }\neq m}^{M}G_{m\leftarrow m^{\prime }}\left(g_{\cdot m^{\prime }};\varphi_{m\leftarrow m^{\prime }}\right)$ means the dependent genetic effect of module *m*, which is also a time-varying function, produced by the influence of other modules on it, *φ_m_* and $\varphi_{m\leftarrow m^{\prime }}$are parameter sets that fit the above 2 parts, respectively. Finally, we utilize the fourth-order Runge-Kutta algorithm to solve the simplified nonlinear differential equation and compute the directional weighted interactions between each pair of modules. Figure 1 illustrates the workflow of the genome-wide multilayer networks that mediate complex dynamic traits and Fig. S1 depicts the analysis and computation flow chart; the detailed descriptions can be seen in the “Analysis and calculation process” section in the Supplementary Materials.

## Results

### Growth curve fitting

**Fig. 2.**
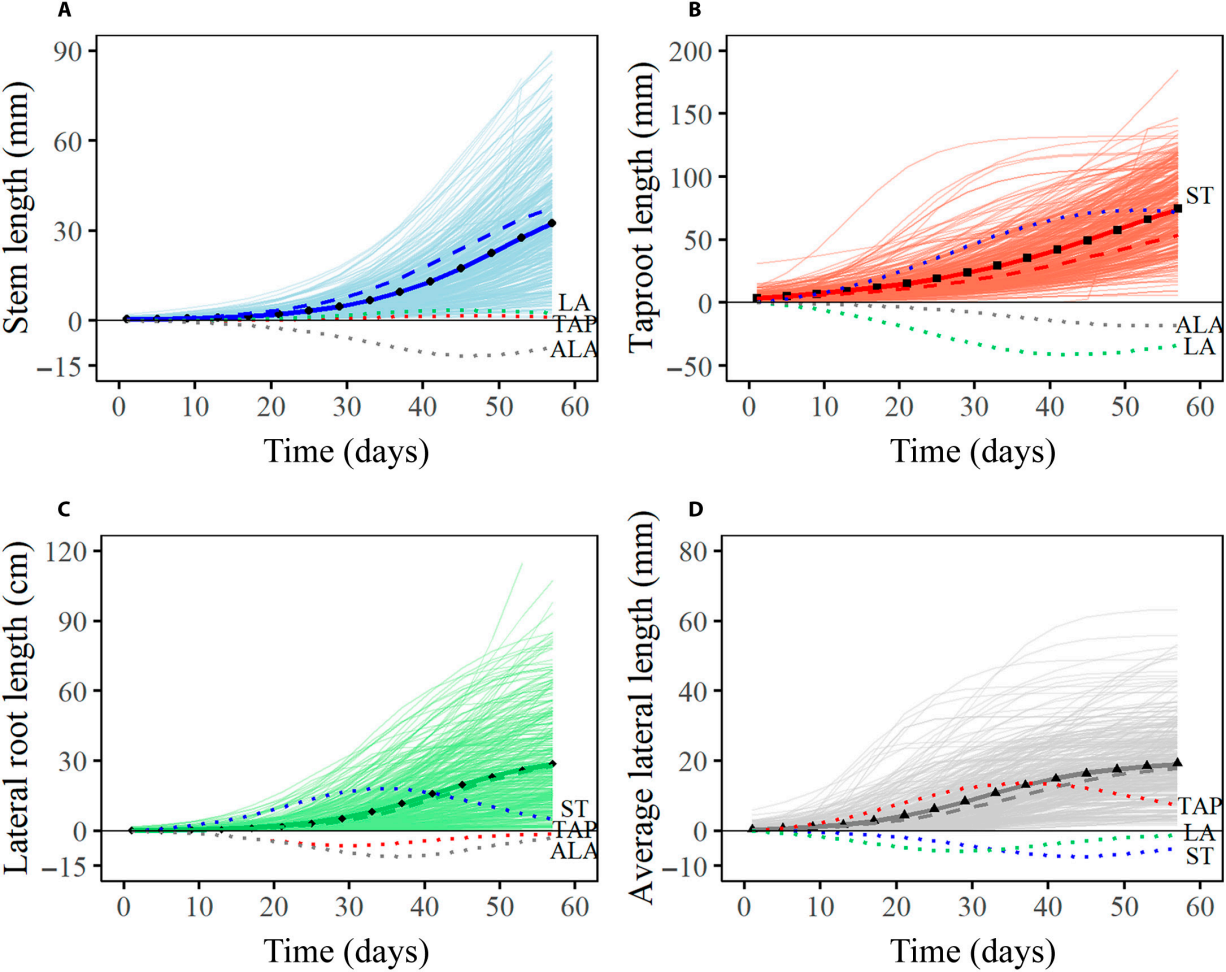
Growth curves of (A) stem length (ST), (B) taproot length (TAP), (C) lateral root length (LA), and (D) average lateral root length (ALA) of *Populus euphratica*. Phenotypic observations of ST, TAP, LA, and ALA of all seedlings are indicated by thin solid lines, corresponding to blue, red, green, and gray, respectively. Average growth values (black scatter) are fitted with MDIM (thick solid lines). The fitting curve of each trait is composed of independent growth (thick broken lines) and dependent growth on the other 3 traits (thick dot lines).

**Fig. 3.**
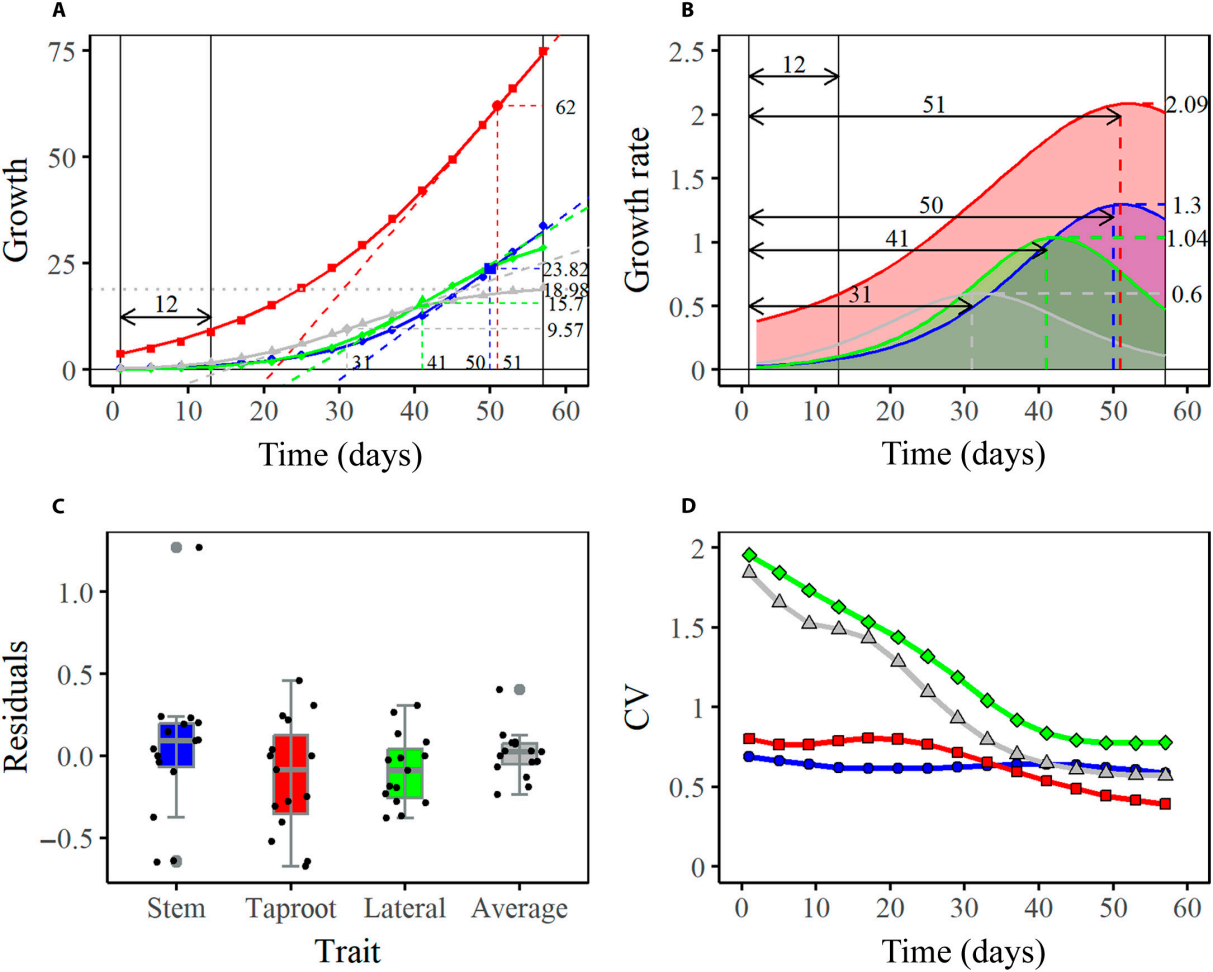
Growth curve fitting and coefficient of variation analysis. (A) Average fitting curves and (B) growth rate curves of stem length (blue), taproot length (red), lateral root length (green), and average lateral root length (gray). (C) Combination of scatterplot and boxplot of residual values. (D) Curves of CV values.

Patterns and extent of trait interactions within organisms vary over developmental time. Compared with many classical equations that only capture the overall growth variation of one trait, such as Richards [55] and the Korf equation [56], MDIM fits the average growth curve of each trait well and also takes into account the interactions among 4 traits (Table S2; Fig. 2), including ST, TAP, LA, and ALA. The non-linear least square method was applied to estimate parameters (Table S1). Through statistical evaluation, the residual sum of squares (RSS < 3), the coefficient of determination (*R*^2^ > 0.99), and the adjusted *R*^2^(>0.99) all indicated satisfactory fitting results. The residuals are randomly distributed above and below zero in Fig. 3C, which means good robustness of MDIM. Four phenotypic traits exhibit distinct time-varying features in Fig. 3A: taproot grows better than the other 3 traits; the gap widens over time and reaches the maximum at 57 days. In the early stage of cultivation (0 to 12 days), the taproot shows important growth while the others almost do not grow. This pattern may meet the growth requirements of plants to root in the soil for enough water and nutrition. Furthermore, the lateral root originates from the pericycle of the taproot, and it starts to grow gradually only when the taproot develops to a certain extent. All traits have similar trends of change; that is, the phenotypic values increase rapidly at an accelerated rate in the early stage, and then the growth rate decreases after reaching the maximum value. However, there are obvious differences in the maximum growth rate (1.3, 2.09, 1.04, and 0.6) and the time point to peak (50, 51, 40, and 41 days). It is worth noting that the growth rate of ALA is close to 0 around 57 days, indicating that it approaches the asymptotic value while the other traits need to grow at a certain rate for a while. With positive growth rates throughout the process, the value of the maximum growth rate and its time point greatly affect the growth status of a trait, which can be verified by the growth rate curve of the TAP. The maximum growth rate of the taproot is greatly greater than other traits, and it arrives at the peak value at the latest (51 days), so its growth condition is greatly better (Fig. 3A and B). In addition, considerable phenotypic variation is detected among samples for each trait (Fig. 3D). The coefficient of variation (CV) for each trait shows a downward trend. Maximum values of CV occur on the first day, then the values decrease over time, but they are always greater than 0.3 (Table S3), indicating the great dispersion among different samples. The underlying causes of differences may be that the seedlings carry different alleles, implicating the possible existence of QTLs that regulate woody growth.

Each fitting curve (overall curve) is the summation of the independent growth part $\frac{{\mathrm{dN}}_{j}^{I}}{\mathrm{dt}}=r_{j}\cdot N_{j}\cdot \left(1-\frac{N_{j}}{K_{j}}\right)\;\left(j=1,2,3,4\right)$ and interactive growth part with the other 3 traits$\frac{{{\mathrm{dN}}_{j}}^{D}}{\mathrm{dt}}=$$\sum_{i=1,i\neq j}^{j}\alpha_{j\leftarrow i}{N_{i}}^{s_{j\leftarrow i}}\left(j=1,2,3,4\right)$, which means that the growth of one trait is not only determined by itself, but also influenced by other traits that coexist with it. Interactions among traits of one individual life can produce different effects, such as cooperative effect, antagonistic effect, and neutral effect. Here, when *α*_*j*←*i*_ > 0, the growth of trait *j* is promoted by trait *i*, while *α*_*j*←*i*_ < 0 corresponds to an antagonistic effect, and *α*_*j*←*i*_ = 0 represents no interaction. In Fig. 2A, the dependent growth curve of ALA is always under zero, so ALA has an obvious inhibitory effect on ST all the time. The promotion effects of TAP and LA offset part of the negative influence. In general, the independent growth of ST outperforms its overall growth. For taproot (Fig. 2B), although LA and ALA have negative effects on it, the powerful promotion effect caused by ST makes the overall growth curve higher than the independent growth curve. It verifies that the growth of the above-ground part is greatly helpful for the root system, but there is competition between the taproot and lateral root, and the concrete manifestation is that as the lateral root grows gradually, its inhibitory effect on the taproot also increases. Furthermore, for LA and ALA, the other 3 traits have an overall positive effect on them (Fig. 2C and D).

### Identification of significant QTLs

**Fig. 4.**
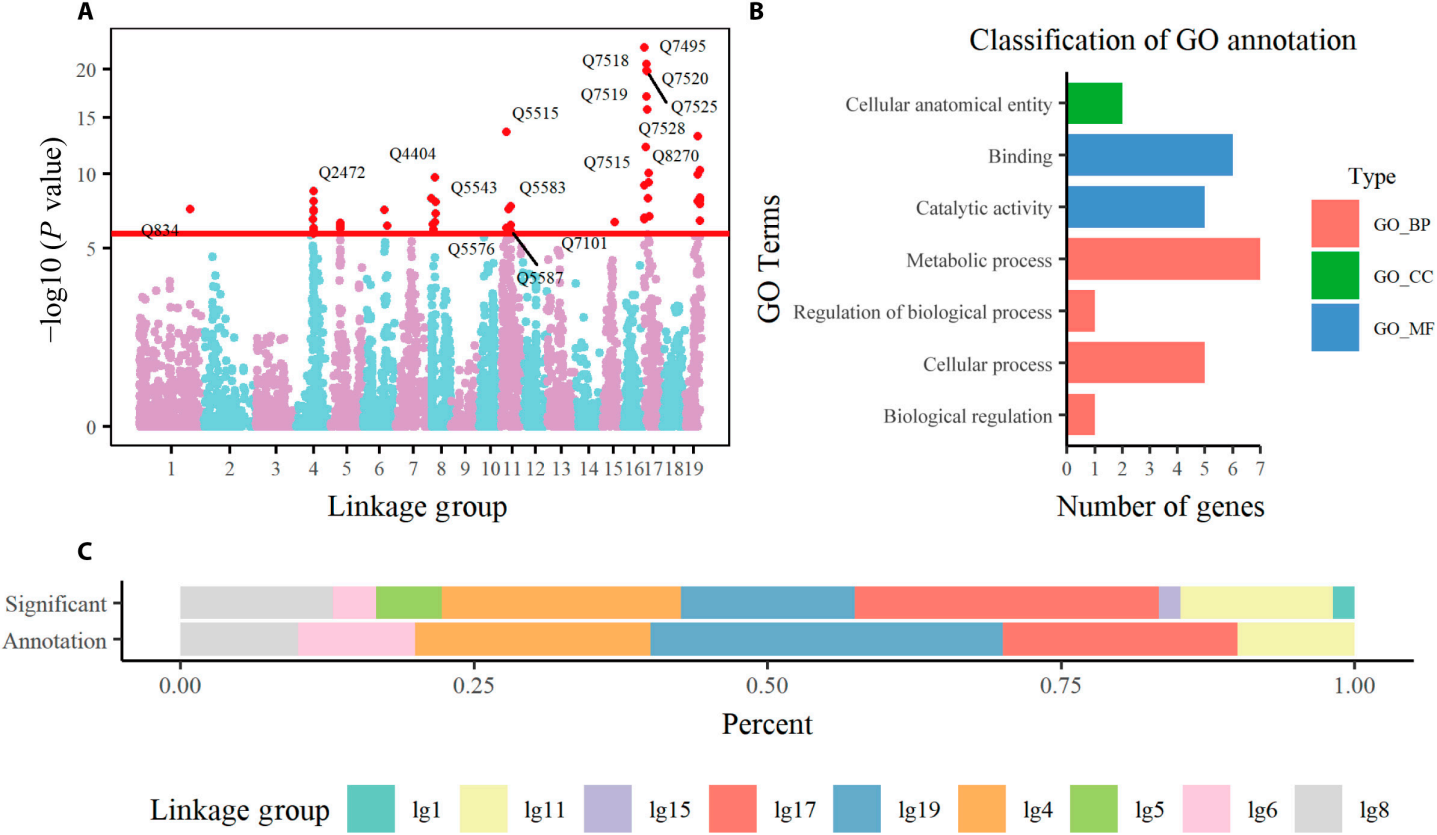
Analysis of identified QTLs. (A) Manhattan plot of *LR* values for 19 linkage groups, and the red horizontal line represents the threshold. (B) includes cellular component (CC), molecular function (MF), and biological process (BP). Bar chart (C) shows the percentage of key QTLs identified by systems mapping and successfully annotated QTLs in each linkage group.

The coordinated process of root–stem growth is closely related to its underlying genetic components, namely, SNPs. By estimating and examining the parameters of different alleles of each SNP, we described how SNPs affected the growth structure of 4 above- and below-ground traits, as well as their competition and cooperation. According to the estimated parameter set of each SNP, the likelihood ratio value *LR* and the *p*-value can be obtained (Fig. S2). FDR corrected the *p*-values of all SNPs and Fig. 4A presents the Manhattan plot of *p*-values over 19 linkage groups. With the critical threshold set to 10^−4^, we identified 54 key SNPs from 8,305 SNPs (labeled as Q1-Q8305), which were sporadically distributed throughout the genome. We referred to the 54 key SNPs as QTLs to distinguish those insignificant SNPs. The number of significant QTLs contained in different linkage groups varies greatly (Fig. 4C). Lg17 has the largest proportion of key QTLs (25.93%) and lg4 accounts for 20.37%, while there is only one QTL on lg1.

To further understand the biological functions of these significant QTLs, their sequences were imported into blast2go for GO functional annotation, in which 10 genes were successfully annotated (Table S4). Some significant QTLs are closely related to functions during plant growth and development. For instance, the gene annotated by Q8269 (hk_hk_1123) on lg19 encodes pentatricopeptide repeat-containing protein At3g04760, and it may be strongly associated with root development and regulation of catalytic activity. Q8267 (lm_ll_2409) was predicted to code putative lysine-specific demethylase JMJ16, which negatively regulates leaf senescence through its activity [57]. Figure 4C displays the distribution of successfully annotated QTLs across linkage groups. Although the number of QTLs identified on lg19 is not the highest, it contains the most annotation information (30.00%), including chromatin organization, protein modification process, etc. Figure 4B shows the distribution of functionally annotated genes in the GO annotation library. The annotation result covers 3 categories, namely, cellular component (CC), molecular function (MF), and biological process (BP), in which BP is mainly involved in the metabolic process and cellular process and MF includes binding and catalytic activity. There were still some QTLs with unclear biological functions. To further enhance the reliability of the results, we conducted sequence alignments with model species (*Arabidopsis thaliana*) to figure out the important functions of homologous genes and their research status in the literature. According to the alignment result and statistical analysis, although the functions of some identified QTLs are currently unclear, they may be homologous to some key genes in *Arabidopsis thaliana*, and these genes have been extensively studied in many articles. For example, Q4371 (lm_ll_7207) may be homologous to gene AT3G07870.1, which encodes F-BOX PROTEIN92 (FBX92), an F-box containing protein. The expression level of FBX92 can affect the size of leaves and cell proliferation rate [58–60]. Q5584 (nn_np_11154) may be homologous to gene AT5G52820.1. AT5G52820.1 encodes a NOTCHLESS homolog, a non-ribosomal protein involved in the maturation and assembly of the 60S ribosomal subunit, whose function has been validated and analyzed in 9 relevant papers.

Our MDIM mapping approach analyzed the genetic control of QTLs with statistically significant effects on multidimensional growth, which was tested by hypotheses based on related growth parameters. Taking the most significant QTL (Q7495, lm_ll_8687) as an example, Fig. S3 illustrates its genetic influence on growth in the stem–root relationship. It is a testcross QTL with 2 alleles lm and ll, corresponding to heterozygote and homozygote, respectively. For most traits, both alleles at this QTL were detected to follow a similar pattern of stem–root interaction. Using the genotypic curves of LA as an example, ST promotes the growth of LA for both alleles, and their growth curves show upward trends followed by downward trends. The growth curve with allele lm appears to have a decreasing trend after about 40 days, while the maximum value of the growth curve with allele ll occurs on about the 50th day. Both the growths of the taproot and average lateral root inhibit the growth of the lateral root. By comparison, the growth curve of taproot with allele lm is more curvaceous than that of taproot with allele ll, as shown by a noted difference in the shape of the growth curves with the 2 alleles. However, for the average lateral root with 2 alleles, the curves show distinct characteristics. Average lateral root growth benefits considerably from taproot growth, while it gains harm from stem growth with allele lm. However, taproot growth and stem growth with allele ll are both favorable for it.

### Multilayer interaction network structure based on genetic effects

**Fig. 5.**
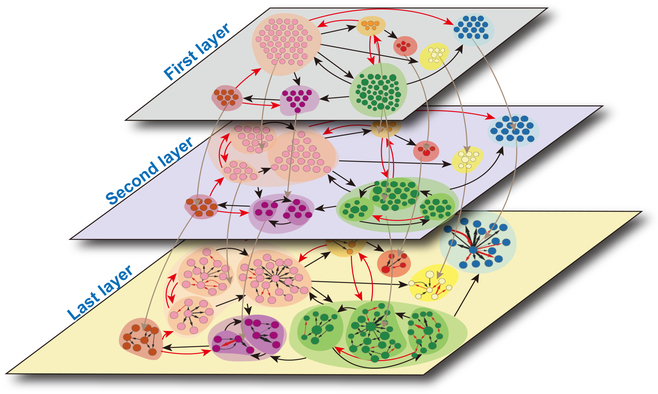
Schematic diagram of the multilayer interactive network model. In the first layer, the whole genome is a large-scale network composed of modules detected by functional clustering. Ellipse shadows with different colors stand for modules that include SNPs with similar dynamic patterns. There are activation (red link) and inhibition (black link) between modules. In the second layer, each module in the first layer is divided into sub-modules with connections. As an example, the pink module contains 3 sub-modules with links. In the last layer, each sub-module representing a small network community contains closely linked variables that are less closely linked with variables from other modules. A small circle within the sub-module represents a minimum unit, i.e., an SNP.

As we all know, the ecosystem on earth forms an intricate, multilevel web, which is composed of many communities distributed in various locations, and there are communications and connections among the communities. Each community contains lots of populations, which can be subdivided into individuals. Learning from this pyramid-like hierarchical structural feature, we built a multilayer interactive network to explore the genome-wide genetic structure. The whole genome was regarded as the ecosystem, divided into communities according to the dynamic characteristics of genetic effects; communities were further subdivided into populations, and populations consisted of some SNPs (Fig. 5). Modularity theory [61,62] manifests that in a huge system composed of multiple modules, the nodes within the module are closely connected, while the modules are relatively sparse. Multiple modules are analogous to communities, just as the connections and exchanges among communities are not as close as those of the same communities. Constructing a multilayer interactive network comprehensively revealed a complete picture of pleiotropic and epistatic genetic architecture in growth traits. From a horizontal perspective, interacting modules constituted a complex network. From a vertical perspective, these modules were further divided into several sub-modules and even sub-sub-modules.

**Fig. 6.**
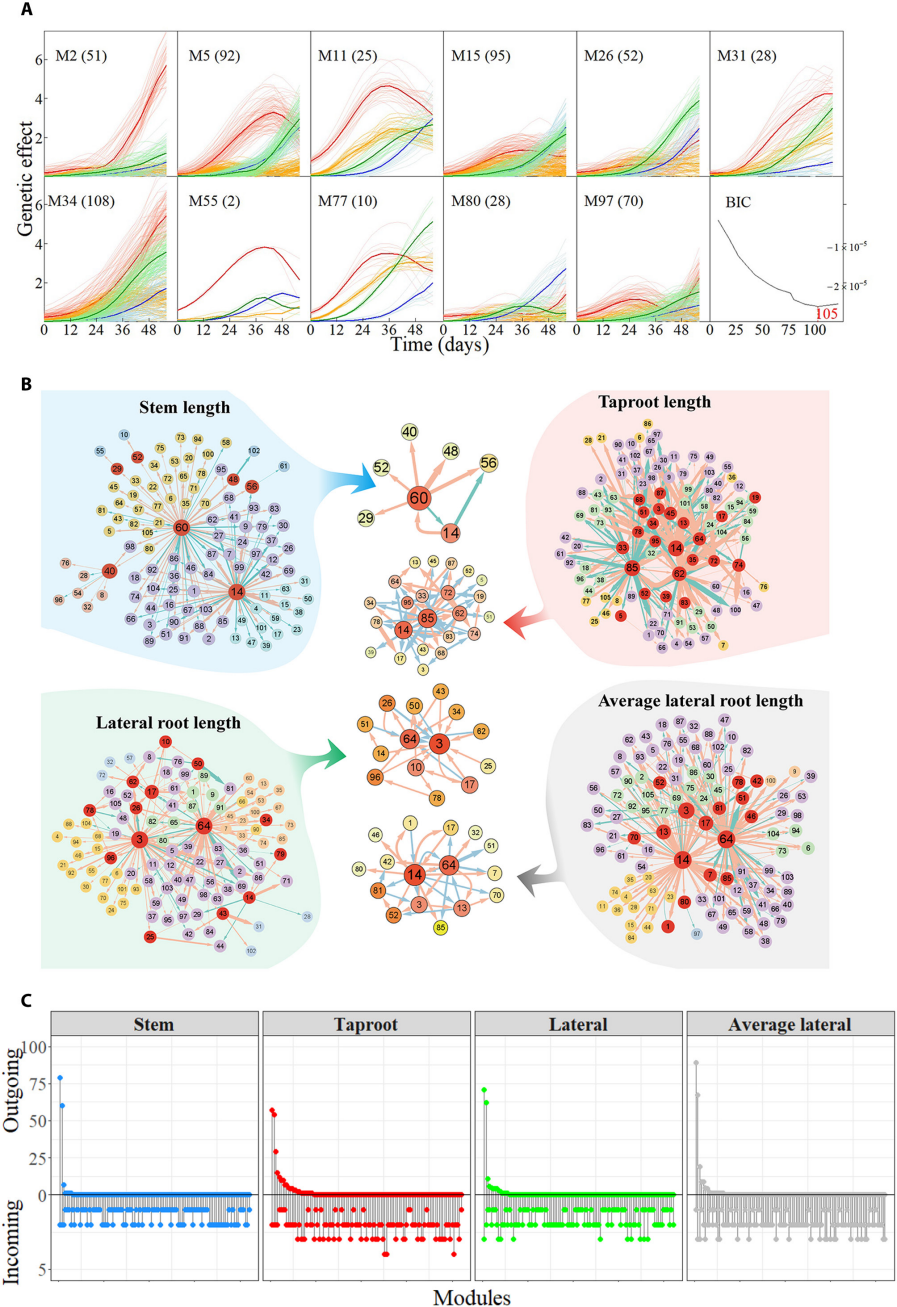
Modules and networks of genetic effects. (A) Genetic effect curves of SNPs in 11 representative modules on stem length (blue line), taproot length (red line), lateral root length (green line), and average lateral root length (orange line) chosen from 105 modules; BIC curve shows that the optimal number of modules is 105. (B) Genetic networks of 105 modules for 4 above- and below-ground traits; there are activation (pink arrowed lines) and inhibition (blue arrowed lines) among modules, with the thickness proportional to the intensity of regulation. (C) The distribution of the number of outgoing modules (upper part) and incoming modules (lower part) of the networks for 4 traits. The *x*-axis is obtained by arranging 105 modules in descending order according to the number of their outgoing modules.

Using multivariable functional clustering, we classified the genetic effects of 4 above- and below-ground traits of 8,305 SNPs into different modules according to the similarity of their effect curves. BIC values showed that the optimal number of modules was 105. The 105 identified modules (labeled as M1 to M105) display diverse patterns of genetic effects (Fig. S4). Figure 6A depicts 11 representative modules. Within a module, the genetic effect curves for the same trait have similar temporal patterns, but different modules exhibit different characteristics. Some modules, including M26 and M31, have great impacts on the stem, but the genetic effects on stem growth of some modules including M2 are always low. In M97, the genetic effects on stem growth are always on the rise. These patterns of genetic effects reflect the diversity of the genetic structure of the synergistic and competitive growth of below-ground roots and above-ground stem.

To figure out the connections among 105 modules, we calculated the mean effect value of each module to construct an interactive genetic network of modules (Fig. 6B). Each module corresponds to a node in the network graph, and there are different degrees of activation or inhibition among nodes. If a module activates or inhibits the other, the module can be called an outgoing module; conversely, an incoming module represents that the module is activated or inhibited by other modules. Outgoing modules tend to be concentrated on a few modules (Fig. 6C); that is, only several highly interconnected modules dominate the genetic networks, such as in the genetic network of ST, where M14, M40, and M60 regulate almost all other modules in the interactive genetic network. It is worth noting that M60 plays the most important regulatory role, which regulates nearly 80 modules, accounting for 74.07% of the total modules. M14 also regulates a large portion of modules and plays a secondary regulatory role; 60 modules regulated account for 57.14%. M40 has a certain regulation on a small part of modules, about 0.07% of the total modules. Incoming modules are uniformly distributed among modules, ranging from 1 to 4. These incoming modules receiving activation or inhibition may perform functions relying on the regulation of other dominant modules. The aforementioned characteristics are also reflected in the other 3 interactive networks, including TAP, LA, and ALA (Fig. 6C). We noticed that M14 belongs to outgoing modules in all 4 networks and regulates a large number of modules in the genetic networks of stem, taproot, and average lateral root, varying from 50 to 90. However, the ability of M14 in the network of the lateral root is relatively minor, with only 5 modules. It is interesting to see that SNPs in M14 perform important biological functions during the development of the plant. As an example, Q1306 (nn_np_3775) may participate in encoding PHD finger protein ALFIN-LIKE 4 (LOC105140713), which has ubiquitin-mediated proteolysis and is involved in biological processes such as endosperm development, plant tissue development, nitrogen compound metabolism, and cellular macromolecular biosynthesis and metabolism [63]. On the other hand, some dominant modules in a network, such as M3, may become secondary modules in other networks. To be specific, M3 plays the main role of regulation in the network of LA, which only belongs to the incoming module in the networks of the other 3 traits. Extracting all outgoing modules and their links from the interactive genetic network of 105 modules, we found that none of the outgoing modules is isolated, and they are closely related to each other. We further discovered some special interactive pathways from the relationship among outgoing modules. For example, in the outgoing module network of TAP, M85 and M62 activate each other to form a mutually beneficial relationship, which means that they belong to both the outgoing module and the incoming module. Similar interactive pathways also exist in Fig. 6B, such as M14↔M17, M3↔M64, and so on. There is only one interaction type (M14↔M60)—parasitic relationship in the network of ST. For details, M14 positively affects the expression of the genetic effects of M60 while M60 inhibits M14. It is worth noting that we observed a long, continuous, and closed-loop path of interaction, i.e., M3→M96→M78→M10→M17→M3. The activation from M3 is transmitted through several other modules and fed back to M3 itself, which constitutes a closed loop.

**Fig. 7.**
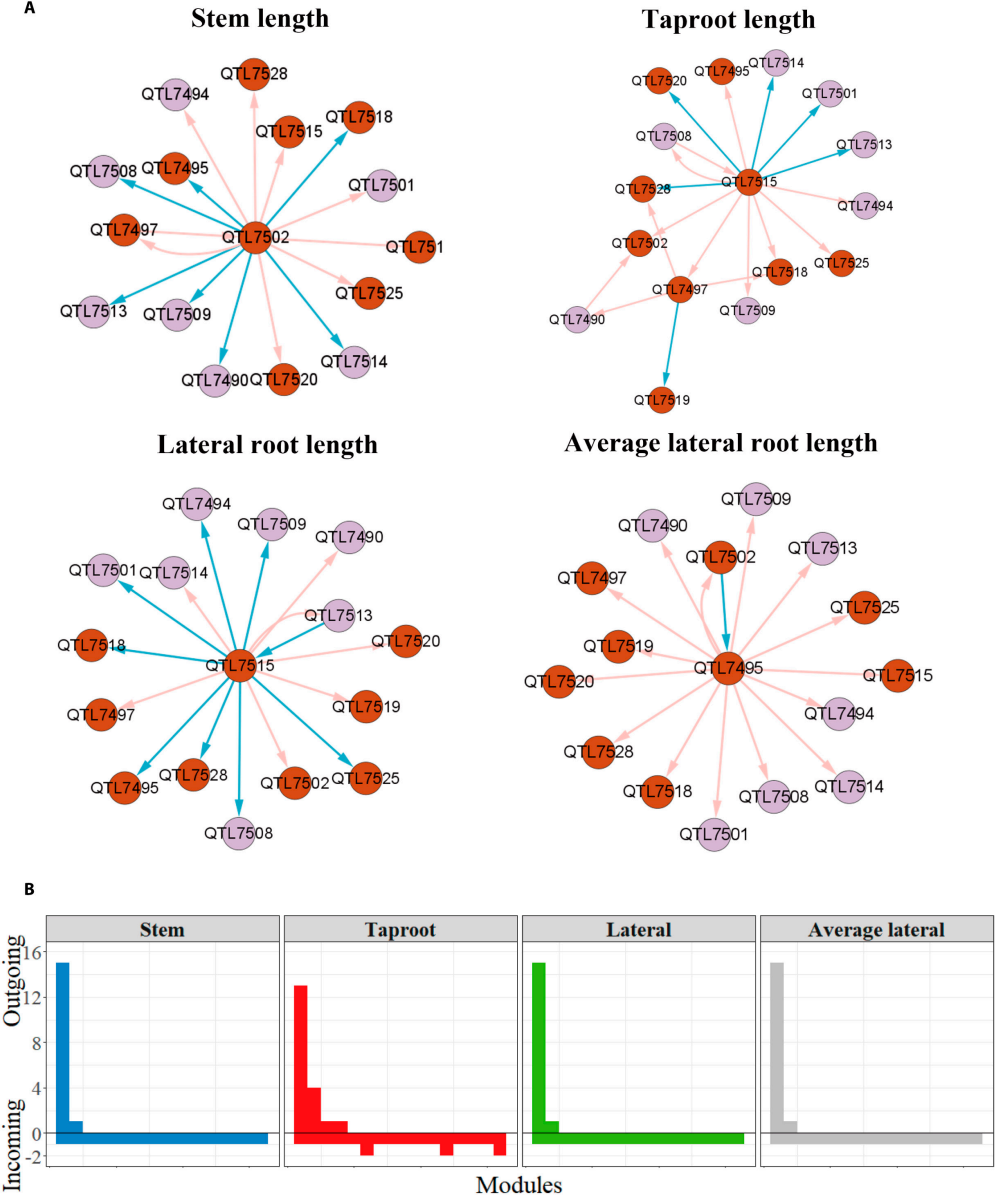
Modules and networks of genetic effects of M24. (A) Genetic effect networks of 4 above- and below-ground traits. Each node represents an SNP or QTL. The directed arrowed lines between nodes represent the interaction relationship, the green one represents the inhibitory effect, the red one represents the promoting effect, and the thickness of the edge is proportional to the strength of the interaction. The brown node represents QTL. (B) The distribution of the number of outgoing modules and incoming modules of 4 traits across SNPs is given.

The 54 significant QTLs detected by multivariate systems mapping based on MDIM are scattered in the 17 modules of the first-layer genetic network. Most QTL-containing modules tend to be influenced by other modules rather than exert influence on other modules; thus, most modules belong to incoming modules in Fig. 6C. This phenomenon suggests that QTLs obtained by our mapping approach may be in a downstream position, and its biological function is indirectly regulated by other modules. We took M24 as a representative, which contains the most QTLs of 17 QTL-containing modules, and then constructed the genetic effect network of the second layer to describe the connections among SNPs in M24. In the genetic effect network organization of the second layer (Fig. 7), each module has only one SNP; that is, each node represents one SNP. We found that only a small part of modules dominates the network, regulating all other nodes, and most nodes are regulated by a small number of modules, belonging to incoming modules. This phenomenon is similar to the first-layer network (Fig. 6). In general, the dominant QTLs are different in the genetic networks of 4 traits. The predominant node Q7502 (hk_hk_2650) in the ST network is a minor node influenced by other nodes in the other 3 networks. Nevertheless, different genetic networks may have the same dominant nodes. We captured such a predominant node Q7515 (lm_ll_8941) in the second-layer networks of the TAP and the LA (Fig. 7A). Different from the first-layer network, these predominant nodes are significant QTLs. We can also find that there are mutually regulated node pairs in each network; in the network of ST, Q7502 and Q7497 (hk_hk_1174) activate each other. In the network of LA, Q7515 has a certain positive effect on Q7513 (lm_ll_11245), while Q7515 inhibits Q7513.

We selected 3 QTLs from M24 to clarify their inherent genetic effects and the impact from other QTLs (Fig. S5). On the one hand, by observing the performances of different QTLs in the network of one trait, such as the stem network, we found that Q7518 (lm_ll_12477) has a dramatic effect on stem growth, and is affected by Q7502, which exerts a negative effect. Therefore, the net genetic effect of Q7518 is inferior to the independent genetic effect. The situation of Q7490 (lm_ll_8106) is similar to that of Q7518. Specifically, the inhibitory effect produced by Q7502 makes the curve of the net genetic effect of Q7490 lower than the curve of the independent genetic effect, but the rangeability of the genetic curves is relatively smaller than the genetic curves of Q7518. By comparison, Q7502 is slightly affected by other QTLs; therefore, the net genetic effect curve of Q7502 almost overlaps with the independent genetic effect curve. On the other hand, by observing the performances of one QTL in different networks, we found that Q7518 is downregulated by Q7502 in the ST network. In contrast, Q7518 is upregulated by Q7495 in the ALA network, and this facilitation occurs after approximately 12 days. In the taproot network, the expression of Q7518 is influenced by Q7497 and Q7515 at the same time. The genetic effect curves of Q7495 and Q7515 show opposite trends, but the effect produced generally inhibits the expression of Q7518. Gene enrichment analysis showed that Q7497 is involved in coding G-type lectin S-receptor-like serine/threonine-protein kinase At4g27290 isoform X2, which plays an important role in signal transduction in and out of cells. Q7497 is further associated with some important biological functions, including the protein modification process, transferase activity, catalytic activity, and so on. These examples all show that the overall genetic effect of a QTL is the combined result of its intrinsic capacity and the indirect regulation of other QTLs, so it is important to study the genetic mechanisms for complex traits from the perspective of the genome-wide genetic network.

### Computer simulation

To verify the reliability of our model, we conducted numerical experiments by mimicking the real growth status of *P. euphratica*. Four root–stem phenotypes interact with one another in a competitive or cooperative manner.

The parameter set of MDIM and the SAD (1) parameters of the covariance structure for each allele on identified key QTLs were used as true values to simulate phenotypic values and allele data. Here, we chose the testcross QTL on lg17 (lm_ll_8687) as a representative. We simulated a full-sib family of size *n* = 100 and 345 (same as the real sample size) with heritabilities of *H*^2^ = 0.05 and 0.1, respectively (see the “Simulation experiment” section in the Supplementary Materials for details). Heritability was used to adjust the parameter values of the SAD (1) covariance matrix.

To demonstrate the reliability of the simulation experiments in replacing real-world growth data, we first compared the genetic effects of real data with those of simulated data. Figure S6 visually shows the consistency of the distribution of genetic effects between real data and simulated data under different sample sizes and heritability. The results of simulation experiments can further show the accuracy and reliability of our model. When the heritability and sample size are small (*H*^2^ = 0.05, *n* = 100), the trends of the simulated growth curves show different characteristics from real growth curves. The precision of parameter estimation improves when sample size and heritability increase. In general, as heritability increases from 0.05 to 0.1 or sample size increases from 100 to 345, the simulated growth curves become closer to the real growth curves (Fig. S7). Fréchet distance provides us with a quantitative and intuitive way to measure the similarity of 2 curves [64]. The magnitude of the Fréchet distance is inversely proportional to the similarity of the curves.

$$\begin{array}{c} \delta_{F}\left(P,Q\right)=\underset{\begin{array}{c} \alpha \left[0,T\right]\to \left|0,N\right|\\ \beta \left[0,T\right]\to \left|0,M\right| \end{array}}{\text{min}}\{\;\underset{t\in \left[0,T\right]}{\text{max}}\;d\left(P\left(\alpha \left(t\right)\right),Q\left(\beta \left(t\right)\right)\right)\} \end{array}$$ (20)

where *t* denotes time, and *α*(*t*) and *β*(*t*) represent the corresponding points on 2 growth curves at time *t*, respectively. *d*(*x*, *y*) represents the Euclidean distance between point *x* and point *y*, and *P*(*a*(*t*)) and *Q*(*β*(*t*)) represent the spatial positions of the corresponding points on 2 growth curves at time *t*, respectively. *δ_F_*(*P*, *Q*) represents the Fréchet distance between curve *P* and curve *Q*. Here, we used the Fréchet distance to evaluate the similarities between the simulated growth curves and the real growth curves, and Table S5 presents the result.

Furthermore, to further verify the reliability of the identified results, we also selected 4 QTLs, which were randomly distributed in the linkage groups. The 4 QTLs are Q2441 (lm_ll_9464, lg4), Q4371 (lm_ll_7207, lg8), Q5587 (nn_np_9210, lg11), and Q7520 (lm_ll_11100, lg11). The above simulation experiments were conducted on these 4 QTLs. Of course, to avoid randomness in the results, the simulation experiments on all QTLs were repeated 100 times, and the average values of the results were taken. Most results are consistent with the results on lm_ll_8687, which means that when the sample size or heritability increases, the simulated curve is closer to the actual curve (Figs. S8 to S11).

## Discussion

In this paper, we propose a comprehensive computational framework that integrates the MDIM, multivariate systems mapping, functional clustering, developmental modularity theory, and evolutionary game theory. The framework performs well in excavating significant genes that regulate the dynamic growth of multiple traits as well as interactions among them and inferring a multilayer large interactive network throughout the genome. MDIM quantitatively describes the dynamic growth of 4 stem–root traits, and the overall growth of each trait consists of a self-regulated part and one that is affected by the other 3 co-existing traits. We embed MDIM into the multivariate systems mapping model to identify QTLs that play critical roles in regulating the growth of multiple complex traits and their interactive relationship. Here, we achieve the generalization of systems mapping from bivariate to multivariate. The simulation experiments are conducted with sample sizes of 100 and 345, and heritability of 0.05 and 0.1, respectively. The results of the simulations show that heritability (the proportion of genetic variance in simulated phenotype variance) and sample size can affect the results of the simulation experiments. Specifically, the accuracy of effect estimation improves with heritability and sample size increases. It has a certain reference meaning for selecting varieties according to heritability or adjusting the sample size appropriately in breeding.

The multilayer interactive network model covering the whole genome achieves a breakthrough from 2-dimensional to high-dimensional, which considers 4 above- and below-ground traits. Through functional clustering, the whole genome is divided into multiple modules, and the relationship among genes in modules is closer than that among modules. Variable selection is incorporated to retain links that are most closely related. The top-layer network covers the information of all genes. There are different degrees of activation or inhibition among modules. Each module is a sub-network, which can be further divided into sub-modules. Sub-modules can be further subdivided until the nodes in the network are single SNPs. The number of layers is not static and will be flexibly adjusted according to the size of the whole genome and the characteristics of the data itself. Algorithms including data smoothing, function clustering, variable selection, and differential equation solving can be improved or optimized by applying efficient mathematical or statistical methods to make the model more accurate.

Multivariate QTL mapping combined with genetic effect networks can not only help us understand the directions, patterns (activation or inhibition), and extent of interactions among modules or SNPs, but also figure out the locations and roles of key QTLs and how these insignificant SNPs work in the genetic network. According to our multivariate systems mapping method, one SNP may not be statistically significant in regulating above- and below-ground growth; however, it influences the expression of key QTLs in the genetic network, indicating an indirect effect on the process of woody growth.

Although we only apply the framework to stem–root data for *P. euphratica*, there is a potential extension for considering other traits, to have a more macroscopic and comprehensive understanding of plant growth regulation and genetic mechanisms. We can take into account stem diameter, number of branches, length of branches, the quantitative traits of flowers and leaves, and so on. On the other hand, the model can be applied to analyze multiple traits of other tree species or plants, such as grass. Moreover, the analytical framework is not confined to plants; there are always competitions and collaborations within and among organisms. We can consider internal competition within one organism, such as the height, weight, arm length, and leg length of a human. We can also focus on the competition among individuals, such as many trees competing for soil, and water in the forest, and different bacterial communities competing for survival resources within the same range. In a word, we provide a reliable model framework to describe the intrinsic genetic mechanisms underlying the development of complex traits.

## Data Availability

The computer code and data that support the findings of this study are deposited in GitHub at https://github.com/Lukaiyan/aboveground_belowground or from the corresponding author upon reasonable request.

## Supplementary Materials

Supplementary 1 Iterative optimization strategy based on maximum likelihood estimation Analysis and calculation process Simulation experiment Figs. S1 to S11 Tables S1 to S5
