# Supplementary material for: Genome-Wide Network Analysis of Above- and Below-Ground Co-growth in Populus euphratica
Source: Plant Phenomics. 2024 Jan 5;6:0131. doi: 10.34133/plantphenomics.0131 (PMC10769449; doi:10.34133/plantphenomics.0131)
Supplement: Supplementary 1 — Iterative optimization strategy based on maximum likelihood estimation Analysis and calculation process Simulation experiment Figs. S1 to S11 Tables S1 to S5 [file plantphenomics.0131.f1.docx]

**Iterative optimization strategy based on maximum likelihood estimation**

Iterative optimization strategy based on maximum likelihood estimation is used to estimate unknown parameters $\Theta=(\theta,\tau)$ in the model by using the iterative ideas in the EM (Expectation Maximization) algorithm for reference. Suppose that $\vec{y}_{1},\vec{y}_{2},\ldots,\vec{y}_{n}$ is the observed data of *n* samples (trees), $f\left( \vec{y}_{i}|\Theta\right)$ is the joint probability density function (*i*=1,2,…,*n*), in which the parameter set $\Theta$ is unknown. Here, parameter set $\Theta$ is composed of two parts: $\theta$ is a subset of parameter set $\Theta$ which describes the mean vector $\vec{\mu}_{j}$ and $\tau$ is a subset comes from covariance matrix $\Sigma$. The objective of the strategy is to find the optimal parameter set $\Theta$ that maximizes the likelihood function, i.e.,

$$\Theta=\arg\max L\left( \vec{y}_{1},\vec{y}_{2},\ldots,\vec{y}_{n};\Theta\right)$$

where $L\left( \vec{y}_{1},\vec{y}_{2},\ldots,\vec{y}_{n};\Theta\right)$ is the logarithm likelihood function:

$$L\left( \vec{y}_{1},\vec{y}_{2},\ldots,\vec{y}_{n};\Theta\right)=\sum_{i=1}^{n} \log f\left( \vec{y}_{i} | \Theta\right)$$

The strategy consists of several steps, The following are the procedures:

**Step0** Set the terminating condition of the strategy. Set the maximum number of iterations as $M_{max}$ and the maximum error as $E_{max}$.

**Step1** An initial guess of the parameter set $\Theta$. According to the average values of *n* samples, obtain the initial estimation $\Theta^{(0)}=(\theta^{(0)},\tau^{(0)})$ of the parameter set $\Theta=(\theta,\tau)$ as the initial iteration.

**Step2** Iterative optimization.

Suppose that $\Theta^{(k)}=(\theta^{(k)},\tau^{(k)})$ is the parameter set obtained at the *k*th iteration ($k<M_{max}$). Each iteration consists of two steps, optimizing $\theta$ and $\tau$ separately.

1. Optimization of $\tau$.

Fix $\theta^{(k)}$ and perform maximum likelihood estimation on $\tau$. Considering the complexity of the model, it is difficult to solve the problem of maximizing the likelihood function using differentiation. the ‘optim’ function in R helps to optimize the parameter set $\tau$ starting from $\tau^{(k)}$ to make the likelihood $L\left( \vec{y}_{1},\vec{y}_{2},\ldots,\vec{y}_{n};\theta^{(k)},\tau\right)$ as larger as possible. Then $\Theta^{(k)}=(\theta^{(k)},\tau^{(k)})$ moves to $(\theta^{(k)},\tau^{(k+1)})$.

1. Optimization of $\theta$.

Fix $\tau^{(k+1)}$ and perform maximum likelihood estimation on $\theta$. The ‘optim’ function in R to optimize the parameter set $\theta$ starting from $\theta^{(k)}$ to make the likelihood $L\left( \vec{y}_{1},\vec{y}_{2},\ldots,\vec{y}_{n};\theta,\tau^{(k+1)} \right)$ as larger as possible. Then $(\theta^{(k)},\tau^{(k+1)})$ moves to $\Theta^{(k+1)}=(\theta^{(k+1)},\tau^{(k+1)})$.

During the optimization process, the differential equations in MDIM is solved using the fourth-order Runge-Kutta algorithm.

**Step3** Determine whether to continue iteration. Calculate the change of parameters:

$$\varepsilon=max|\Theta^{(k)}-\Theta^{(k+1)}|$$

If $\varepsilon<E_{max}$ and $k<M_{max}$, continue **Step2**. Otherwise, the algorithm ends and outputs the final parameter set $\Theta$.

**Analysis and calculation process**

(1) Data preparation and input.

Phenotypic data of different traits of plants at sequential points with sufficient samples, as well as high-throughput sequencing data (SNP data) of the plant.

(2) Initialization parameters.

Select appropriate initial parameters before calculation, it depends on the growth characteristics of different plants.

(3) Fitting of equations.

By using the least squares method, the initial parameters of MDIM are iteratively optimized and the fitting results are evaluated based on multiple evaluation criteria.

(4) Identification of significant QTLs.

Embed systems mapping into hypothesis-testing, and conduct hypothesis testing on all SNPs one by one to obtain a series of *p*-values, and screen QTLs based on the *p*-values.

(5) Calculation of genetic effect.

(6) Determination of module quantity.

Calculate the BIC values for different module quantities and select the clustering result with the lowest BIC value.

(7) Construction of the first layer network.

Based on the best clustering result obtained in the previous step, calculate the average genetic effect of each module to construct the first layer network, which contains smoothing data, selecting variables, and solving linear differential equations.

(8) Construction of the next layer network.

It depends on the number of SNPs in a module whether to further divide a module into sub-modules and construct the next layer network.

(9) Construction of the multi-layer network.

Repeat steps 7 and 8 until the module can’t be divided. Finish the construction of the multi-layer network.

**Simulation Experiment**

We first randomly select a QTL from the significant QTLs identified by our model. Based on the parameter set of MDIM and the SAD (1) parameters of the covariance structure for each allele on the selected QTL, while considering environmental factors, we simulate phenotypic values of a full-sib family with n seedlings and allele data of one QTL. The simulation of phenotypic values is based on the distribution of phenotypes in population with selected alleles under the current growth environment, while also considering heritability. Apply our model to the simulated phenotype and allele data to obtain new parameter set of this QTL. To avoid the interference of random factors on the experimental results, the above experimental process will be repeated 100 times, and we will take the average values of the 100 results. The final result will be compared with the actual parameter values of this QTL.

Table S1. The estimated parameters and the evaluation information of MDIM.

|  | Stem (mm) | Taproot (mm) | Lateral root (cm) | Average lateral root (mm) |
| --- | --- | --- | --- | --- |
| Independent part | $K_{1}=$49.069 | $K_{2}=102.270$ | $K_{3}=33.019$ | $K_{4}=19.439$ |
|  | $r_{1}=0.106$ | $r_{2}=0.060$ | $r_{3}=0.130$ | $r_{4}=0.114$ |
| Interactive part | $\alpha_{1\leftarrow2}=0.007$ | $\alpha_{2\leftarrow1}=$0.801 | $\alpha_{3\leftarrow1}=0.143$ | $\alpha_{4\leftarrow1}=-0.031$ |
|  | $s_{1\leftarrow2}=-0.059$ | $s_{2\leftarrow1}=0.346$ | $s_{3\leftarrow1}=-0.330$ | $s_{4\leftarrow1}=0.180$ |
|  | $\alpha_{1\leftarrow3}=0.012$ | $\alpha_{2\leftarrow3}=-0.588$ | $\alpha_{3\leftarrow2}=-0.077$ | $\alpha_{4\leftarrow2}=0.143$ |
|  | $s_{1\leftarrow3}=0.373$ | $s_{2\leftarrow3}=0.296$ | $s_{3\leftarrow2}=-0.016$ | $s_{4\leftarrow2}=0.056$ |
|  | $\alpha_{1\leftarrow4}=-0.035$ | $\alpha_{2\leftarrow4}=-0.162$ | $\alpha_{3\leftarrow4}=-0.07$ | $\alpha_{4\leftarrow3}=-0.099$ |
|  | $s_{1\leftarrow4}=0.094$ | $s_{2\leftarrow4}=0.221$ | $s_{3\leftarrow4}=$-0.432 | $s_{4\leftarrow3}=-0.094$ |
| RSS | 2.826 | 1.960 | 0.765 | 0.317 |
| $R^{2}$ | 0.998 | 0.999 | 0.999 | 0.999 |
| Adjusted $R^{2}$ | 0.997 | 0.999 | 0.999 | 0.999 |
| AIC | -9.0217 | -16.628 | -28.822 | -44.5738 |

Table S2. The estimated parameters and the evaluation information of classic growth equations, including Korf equation and Richards equation.

|  | **Korf** | | | | **Richards** | | | |
| --- | --- | --- | --- | --- | --- | --- | --- | --- |
|  | Stem (mm) | Taproot (mm) | Lateral root (cm) | Average lateral root (mm) | Stem (mm) | Taproot (mm) | Lateral root (cm) | Average lateral root  (mm) |
| $A$ | 884.5481 | 1126.829 | 93.1376 | 43.7459 | 685.3806 | 284.7532 | 54.4684 | 21.0424 |
| $k$ | 61.1570 | 18.6233 | 227.5219 | 52.8052 | 0.0141 | 0.0208 | 0.0419 | 0.0900 |
| $B$ | 0.7226 | 0.4720 | 1.3065 | 1.0393 | 0.5605 | 0.1663 | 1.0387 | -7.1672 |
| $m$ | / | / | / | / | 0.9045 | 0.9613 | 0.8360 | 1.4677 |
| *RSS* | 11.6020 | 134.6705 | 2.9869 | 3.0466 | 1.8850 | 3.2262 | 2.8030 | 0.1073 |
| $R^{2}$ | 0.9928 | 0.9824 | 0.9625 | 0.9957 | 0.9988 | 0.9996 | 0.9982 | 0.9998 |
| Adjusted $R^{2}$ | 0.9857 | 0.9648 | 0.9250 | 0.9914 | 0.9977 | 0.9992 | 0.9963 | 0.9997 |
| AIC | 2.1469 | 38.9217 | -18.3815 | -17.9104 | -23.1118 | -15.0512 | -17.1607 | -66.1032 |

Table S3. CV values at different timepoint.

|  | $t_{1}$ | $t_{2}$ | $t_{3}$ | $t_{4}$ | $t_{5}$ | $t_{6}$ | $t_{7}$ | $t_{8}$ | $t_{9}$ | $t_{10}$ | $t_{11}$ | $t_{12}$ | $t_{13}$ | $t_{14}$ | $t_{15}$ |
| --- | --- | --- | --- | --- | --- | --- | --- | --- | --- | --- | --- | --- | --- | --- | --- |
| Stem | 0.69 | 0.66 | 0.64 | 0.62 | 0.62 | 0.62 | 0.62 | 0.63 | 0.63 | 0.64 | 0.64 | 0.64 | 0.62 | 0.61 | 0.59 |
| Taproot | 0.80 | 0.77 | 0.77 | 0.79 | 0.81 | 0.80 | 0.77 | 0.72 | 0.66 | 0.59 | 0.54 | 0.49 | 0.45 | 0.42 | 0.39 |
| Lateral | 1.96 | 1.84 | 1.73 | 1.63 | 1.53 | 1.44 | 1.32 | 1.19 | 1.04 | 0.92 | 0.84 | 0.79 | 0.78 | 0.78 | 0.78 |
| Average lateral | 1.84 | 1.66 | 1.52 | 1.49 | 1.43 | 1.28 | 1.09 | 0.93 | 0.79 | 0.70 | 0.65 | 0.61 | 0.59 | 0.58 | 0.57 |

Table S4. The chromosomal positions and GO functional annotation of significant QTLs detected by systems mapping to affect the growth in the F1 mapping population of *Populus euphratica.*

|  | Marker ID | Linkage Group | Genetic  distances  (cM) | GO term | GO name | Annotation |
| --- | --- | --- | --- | --- | --- | --- |
| Q2461 | lm_ll_11682 | lg4 | 140.71 | P: GO:0015074;  F: GO:0003676 | P: DNA integration;  F: nucleic acid binding | reverse transcriptase domain-containing protein |
| Q2468 | lm_ll_11931 | lg4 | 141.72 | P: GO:0015074;  F: GO:0003676 | P: DNA integration;  F: nucleic acid binding | reverse transcriptase domain-containing protein |
| Q3677 | lm_ll_12965 | lg6 | 193.11 | P: GO:0036211;  F: GO:0016787;  F: GO:0140096 | P: protein modification process;  F: hydrolase activity;  F: catalytic activity, acting on a protein | probable protein phosphatase 2C 40 isoform X1 |
| Q4230 | lm_ll_12636 | lg8 | 3.96 | P: GO:0006355;  F: GO:0016787 | P: regulation of transcription, DNA-templated;  F: hydrolase activity | integrase |
| Q5576 | nn_np_9673 | lg11 | 73.58 | F: GO:0003723 | F: RNA binding | heterogeneous nuclear ribonucleoprotein F |
| Q7496 | lm_ll_2545 | lg17 | 3.01 | P: GO:0015074;  F: GO:0003676;  F: GO:0008270 | P: DNA integration;  F: nucleic acid binding;  F: zinc ion binding | gag-pol polyprotein |
| Q7497 | hk_hk_1174 | lg17 | 3.01 | P: GO:0036211;  F: GO:0016740;  F: GO:0140096;  C: GO:0005886 | P: protein modification process;  F: transferase activity;  F: catalytic activity, acting on a protein;  C: plasma membrane | G-type lectin S-receptor-like serine/threonine-protein kinase At4g27290 isoform X2 |
| Q8267 | lm_ll_2409 | lg19 | 100.51 | P: GO:0006325;  P: GO:0036211;  F: GO:0016491; F: GO:0016740;  F: GO:0140096; C: GO:0005634 | P: chromatin organization;  P: protein modification process;  F: oxidoreductase activity;  F: transferase activity; F: catalytic activity, acting on a protein;  C: nucleus | putative lysine-specific demethylase JMJ16 |
| Q8269 | hk_hk_1123 | lg19 | 102.43 | F: GO:0005515; F: GO:0016740 | F: protein binding;  F: transferase activity | pentatricopeptide repeat-containing protein At3g04760, chloroplastic-like |
| Q8295 | lm_ll_1936 | lg19 | 120.32 | F: GO:0003677 | F: DNA binding | probable disease resistance protein At4g27220 isoform X1 |

Table S5. Fréchet distance between simulated growth curves and real growth curve with different sample sizes and heritability.

|  | | | n=100 | | n=345 | |
| --- | --- | --- | --- | --- | --- | --- |
|  |  |  | $H^{2}$=0.05 | $H^{2}$=0.1 | $H^{2}$=0.05 | $H^{2}$=0.1 |
| Stem length | heterozygote | total | 0.704459 | 0.83718 | 0.760021 | 0.536493 |
|  |  | ind | 24.17752 | 24.76351 | 25.49763 | 15.81249 |
|  |  | inter | 24.88198 | 25.60069 | 26.25765 | 16.34899 |
|  | homozygote | total | 0.326865 | 0.35685 | 0.348945 | 0.398305 |
|  |  | ind | 10.93044 | 11.07512 | 11.26979 | 8.454728 |
|  |  | inter | 10.65155 | 10.89546 | 11.05887 | 8.386893 |
| Taproot  length | heterozygote | total | 0.892758 | 0.579419 | 0.880571 | 0.340944 |
|  |  | ind | 10.11684 | 8.575716 | 7.862072 | 1.163268 |
|  |  | inter | 11.00311 | 9.155135 | 8.742643 | 1.270329 |
|  | homozygote | total | 0.433624 | 0.335026 | 0.506232 | 0.363872 |
|  |  | ind | 3.881442 | 3.520603 | 3.866006 | 3.518303 |
|  |  | inter | 3.68097 | 3.284496 | 3.69042 | 3.403998 |
| Lateral root length | heterozygote | total | 0.546372 | 0.599858 | 0.634574 | 0.379915 |
|  |  | ind | 12.52362 | 10.29234 | 12.18142 | 8.989023 |
|  |  | inter | 12.47881 | 10.32661 | 12.0584 | 8.926132 |
|  | homozygote | total | 0.392479 | 0.305615 | 0.40009 | 0.317051 |
|  |  | ind | 13.41673 | 12.5326 | 14.46829 | 11.24377 |
|  |  | Inter | 13.12754 | 12.24842 | 14.17431 | 10.95586 |
| Average lateral root length | heterozygote | total | 0.359009 | 0.255501 | 0.386759 | 0.253091 |
|  |  | ind | 5.686313 | 4.930096 | 7.24513 | 4.700004 |
|  |  | inter | 5.72802 | 4.994541 | 7.236042 | 4.658085 |
|  | homozygote | total | 0.302322 | 0.224224 | 0.299497 | 0.265612 |
|  |  | ind | 2.621306 | 1.175984 | 2.50553 | 0.719463 |
|  |  | inter | 2.409891 | 0.95386 | 2.271925 | 0.807982 |


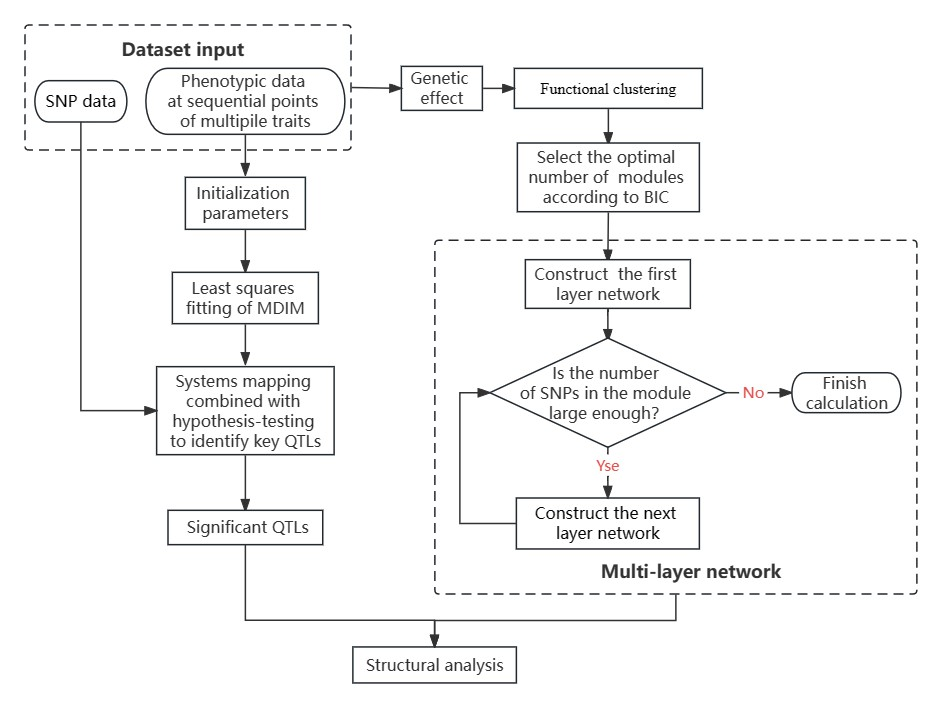


Fig. S1. Analysis and computation flow chart of genome-wide multilayer networks that mediate complex dynamic traits.


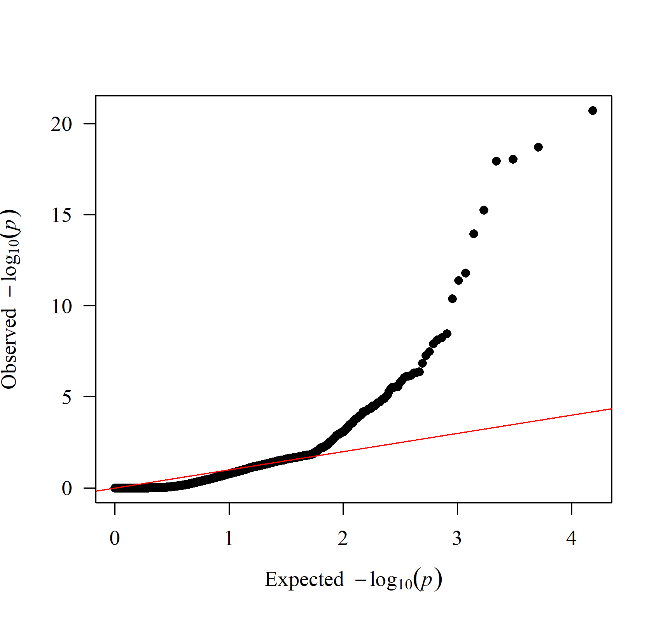


Fig. S2. Q–Q plot of *p*-value characterizes the degree of deviation between the observed distribution and the expected distribution.


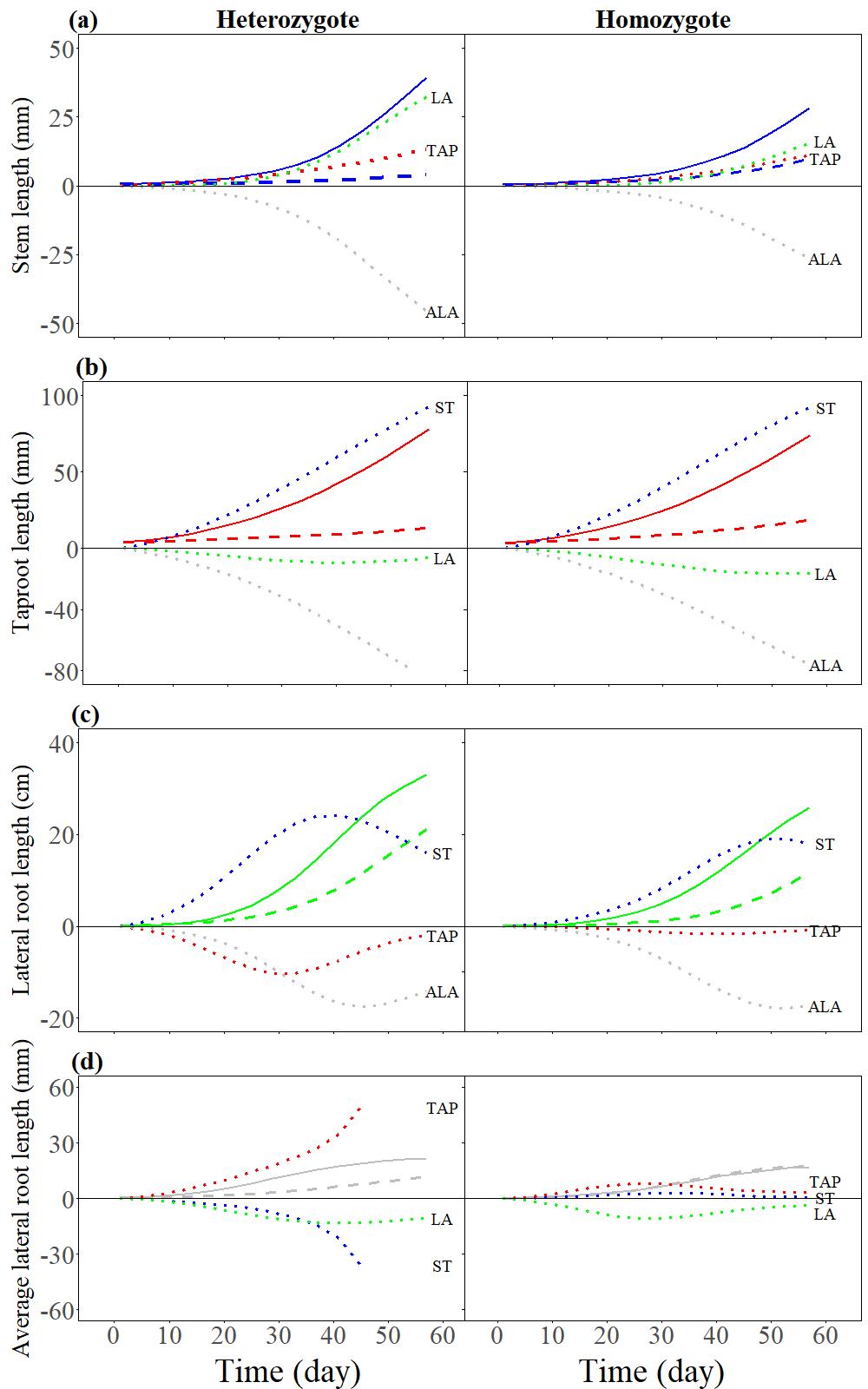


Fig. S3. Genotypic curves of stem length (a), taproot length (b), lateral root length (c) and average lateral root length (d), explained by Q7495 (lm_ll_8687). The overall growth (solid lines) of stem length (ST, blue line), taproot length (TAP, red line), lateral root length (LA, green line) and average lateral root length (ALA, grey line) is contributed by independent growth (broke lines) and dependent growth (dot lines) on the other three traits. Two genotypes are heterozygote lm and homozygote ll.


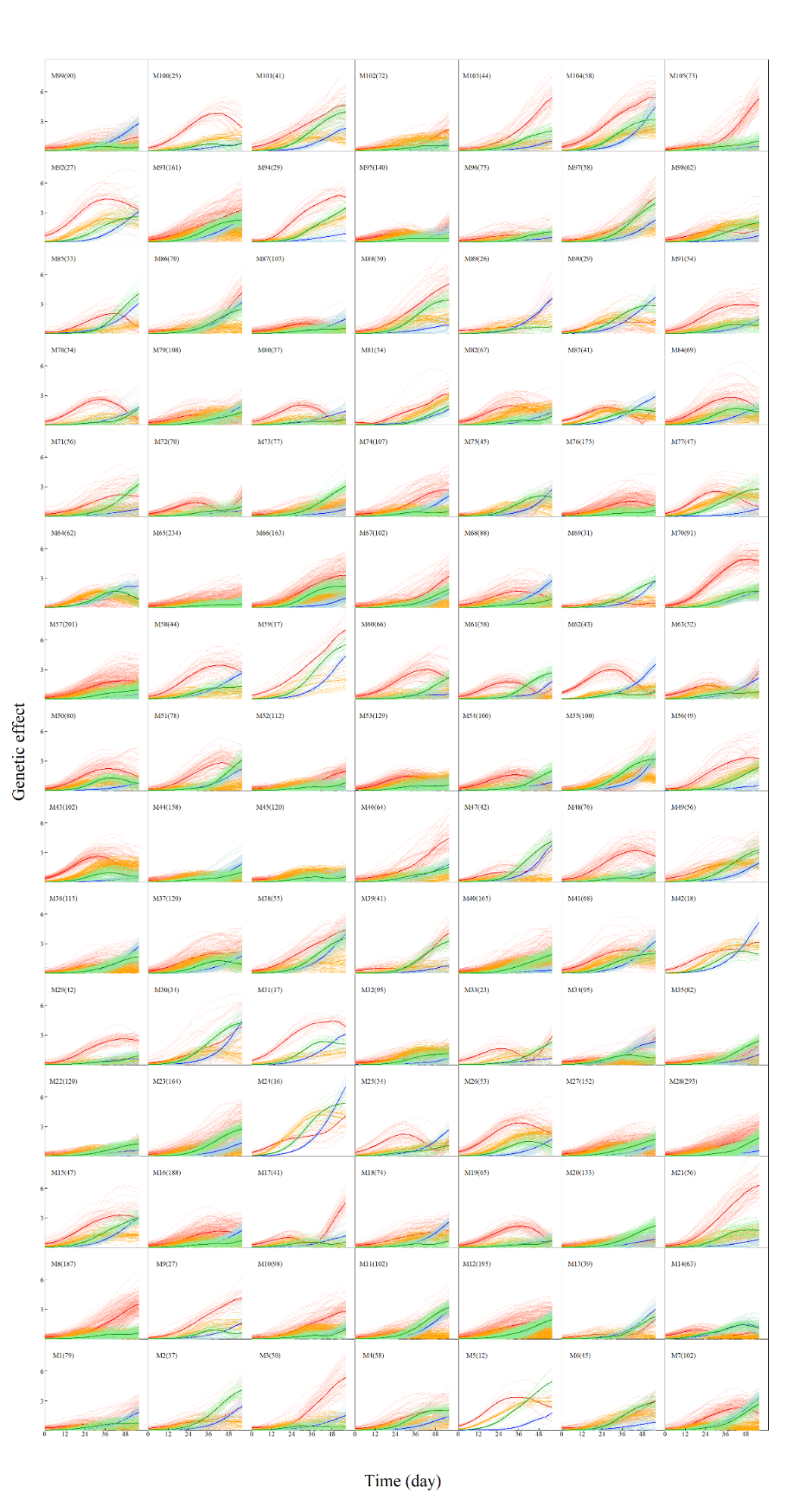


Fig. S4. Genetic effect curves for 105 modules of stem length (blue line), taproot length (red line), lateral root length (green line), and average lateral root length (orange line).


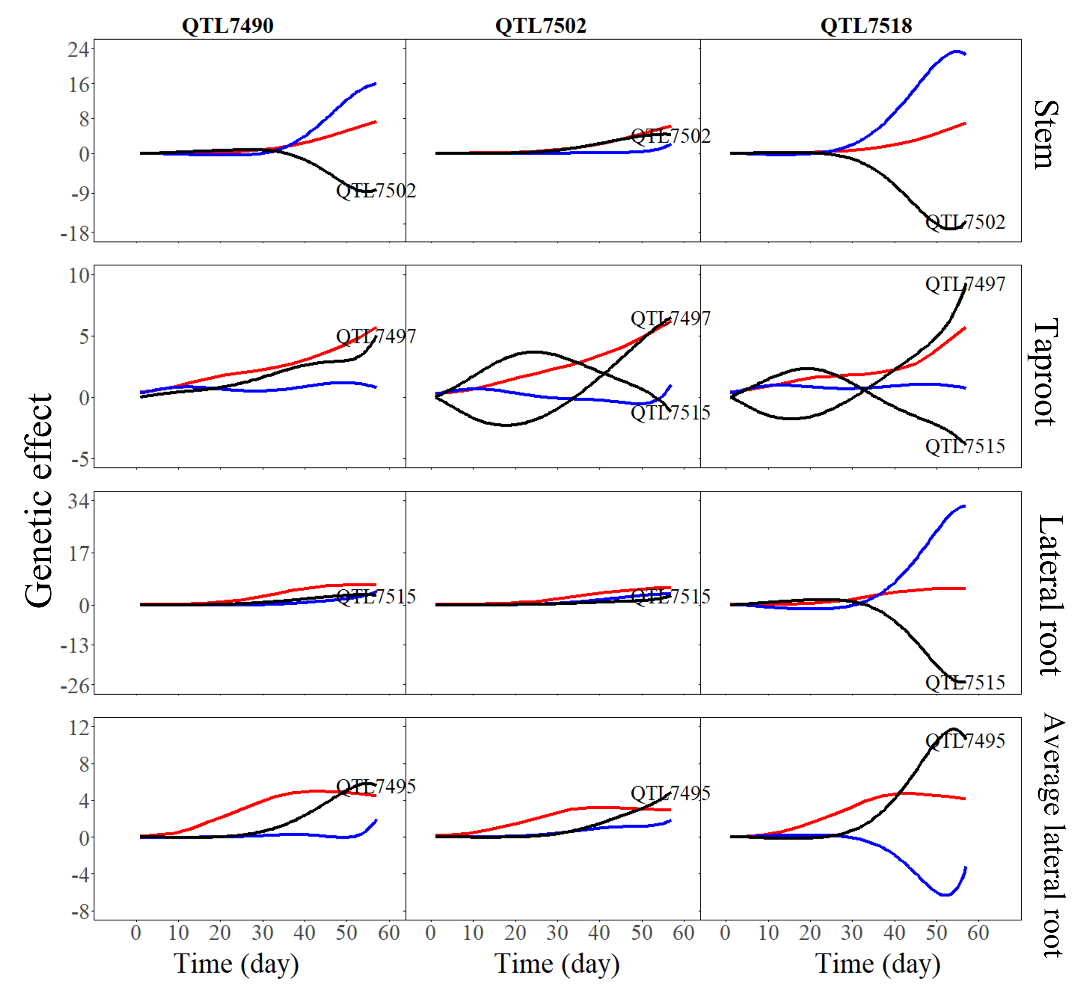


Fig. S5. Genetic effect curves of three QTLs in M24 of stem length, taproot length, lateral root length and average lateral root length. The net genetic effect of a QTL (red line) consists of independent genetic effect (blue) and dependent genetic effects on other QTLs (black line).


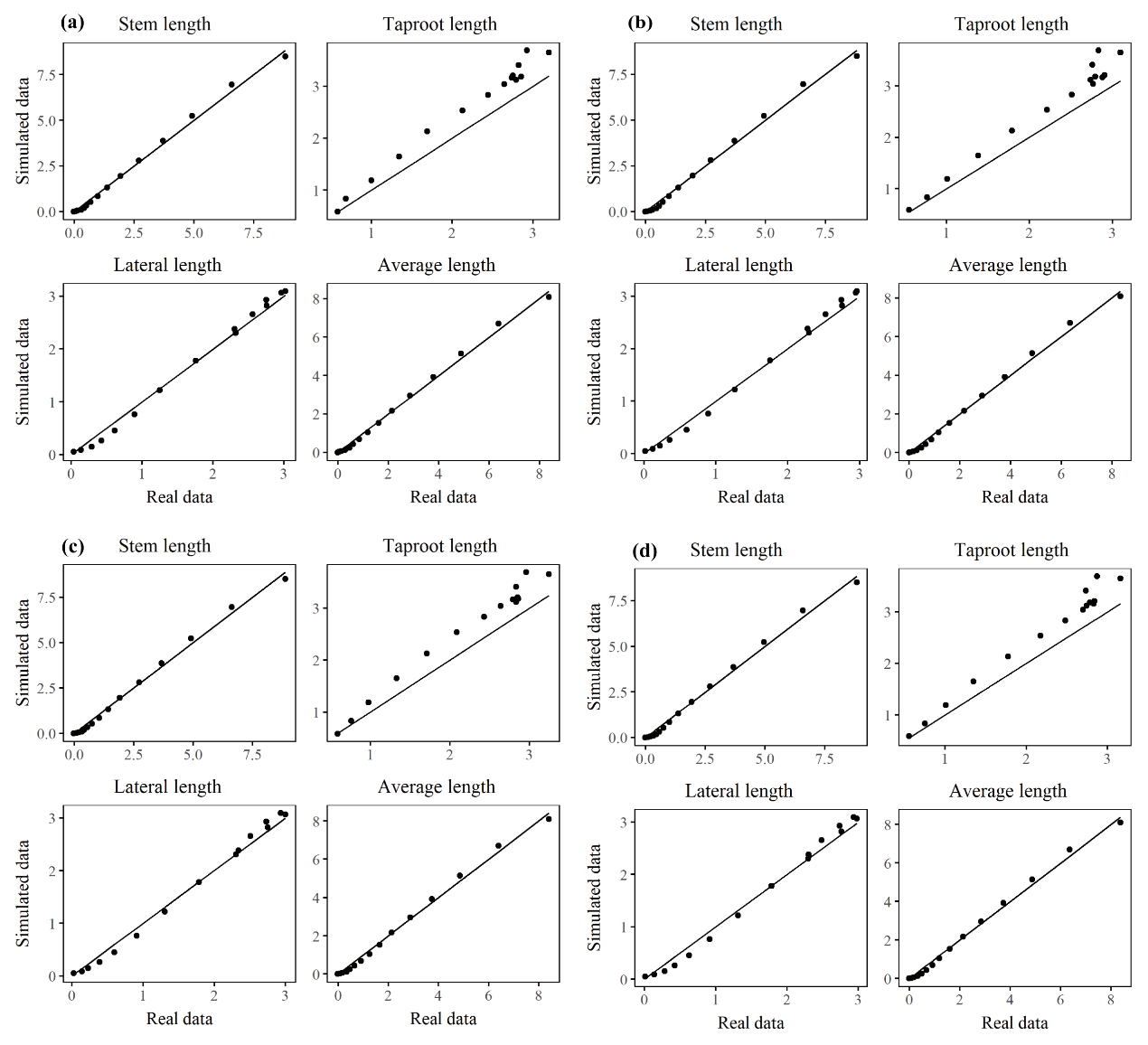


Fig. S6. Q–Q plot of genetic effects characterize the degree of deviation between the real data and the simulated data under (a) the sample size 100 and the heritability 0.05; (b) the sample size 345, and the heritability 0.05; (c) the sample size 100, and the heritability 0.1; (d) the sample size 345, and the heritability 0.1.


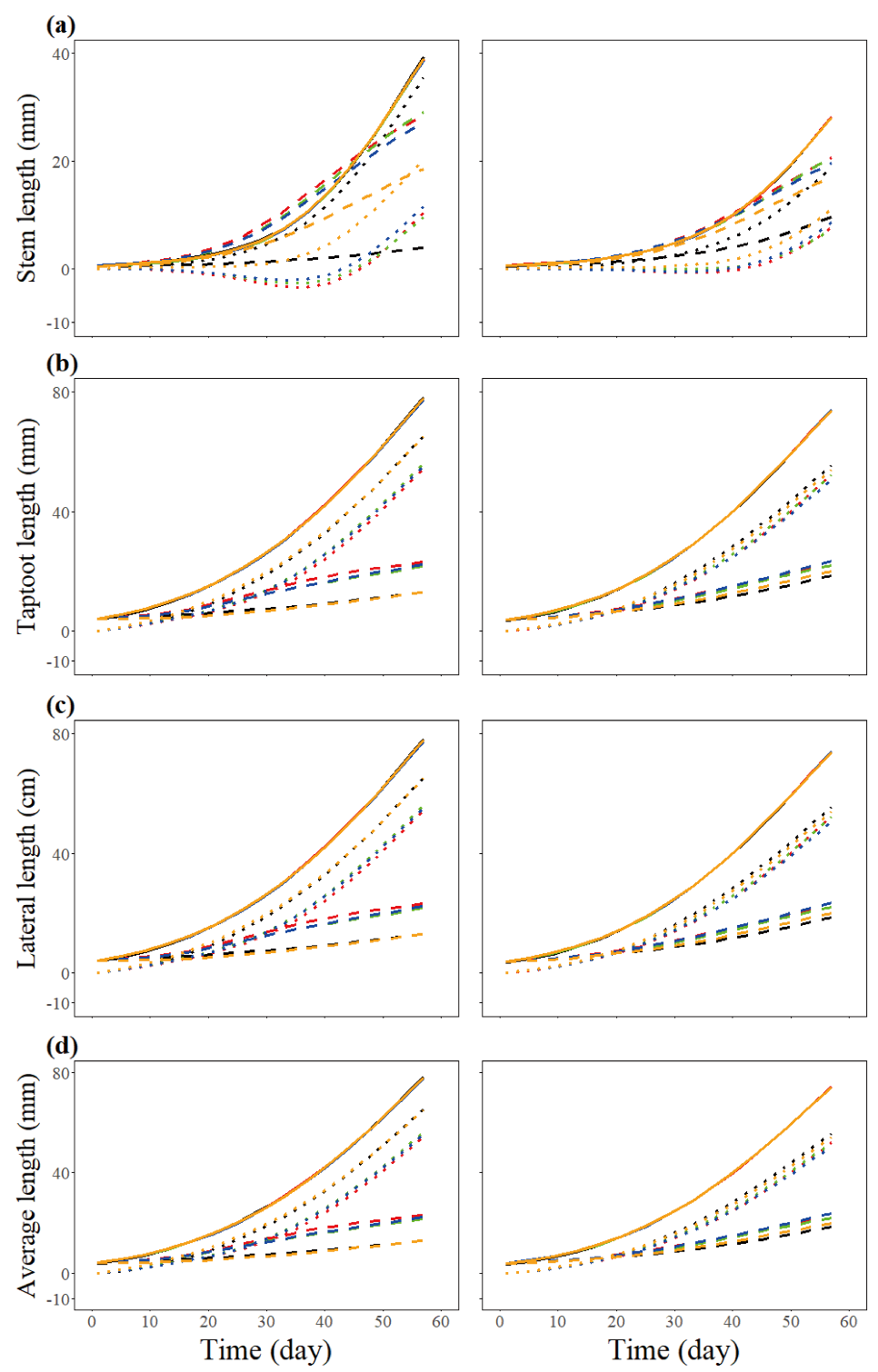


Fig. S7. Simulation results of heritability 0.05 (red lines) and 0.1 (green lines) when the sample size is 100, and simulation results of the heritability 0.05 (blue lines) and 0.1 (orange lines) when the sample size is 345. Simulated growth curves (dotted lines) of heterozygote and homozygote for stem length (a), taproot length (b), lateral root length (c) and the average lateral root length (d) are compared with the real curves (black lines). The solid lines represent the overall growth curves, the broke lines represent the independent growth curves, and the dot lines represent one trait is influenced by the other three traits.


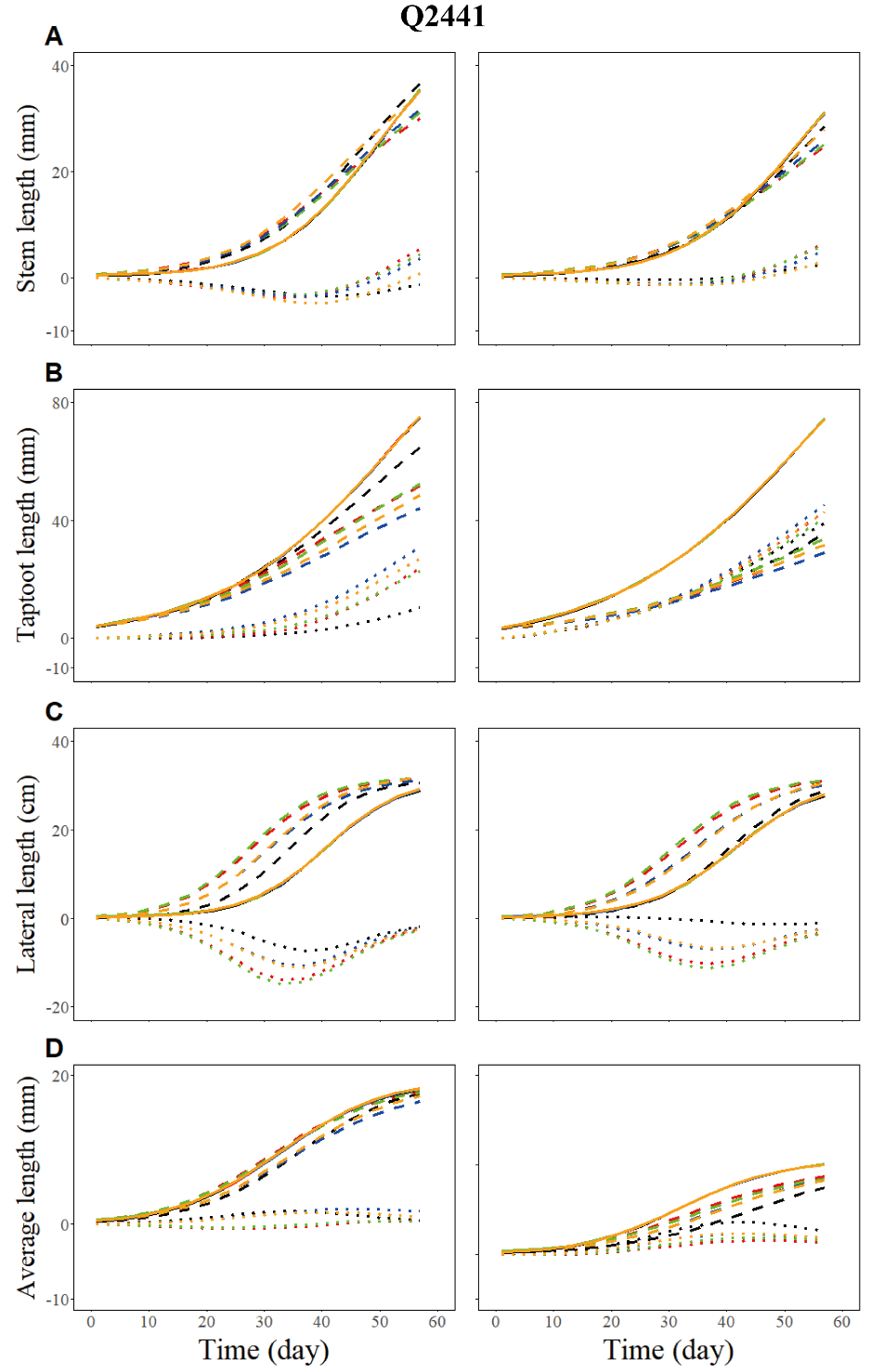


Fig. S8. Simulation results of heritability 0.05 (red lines) and 0.1 (green lines) when the sample size is 100, and simulation results of the heritability 0.05 (blue lines) and 0.1 (orange lines) when the sample size is 345 (Q2441). Simulated growth curves (dotted lines) of heterozygote and homozygote for stem length (A), taproot length (B), lateral root length (C) and the average lateral root length (D) are compared with the real curves (black lines). The solid lines represent the overall growth curves, the broke lines represent the independent growth curves, and the dot lines represent one trait is influenced by the other three traits.


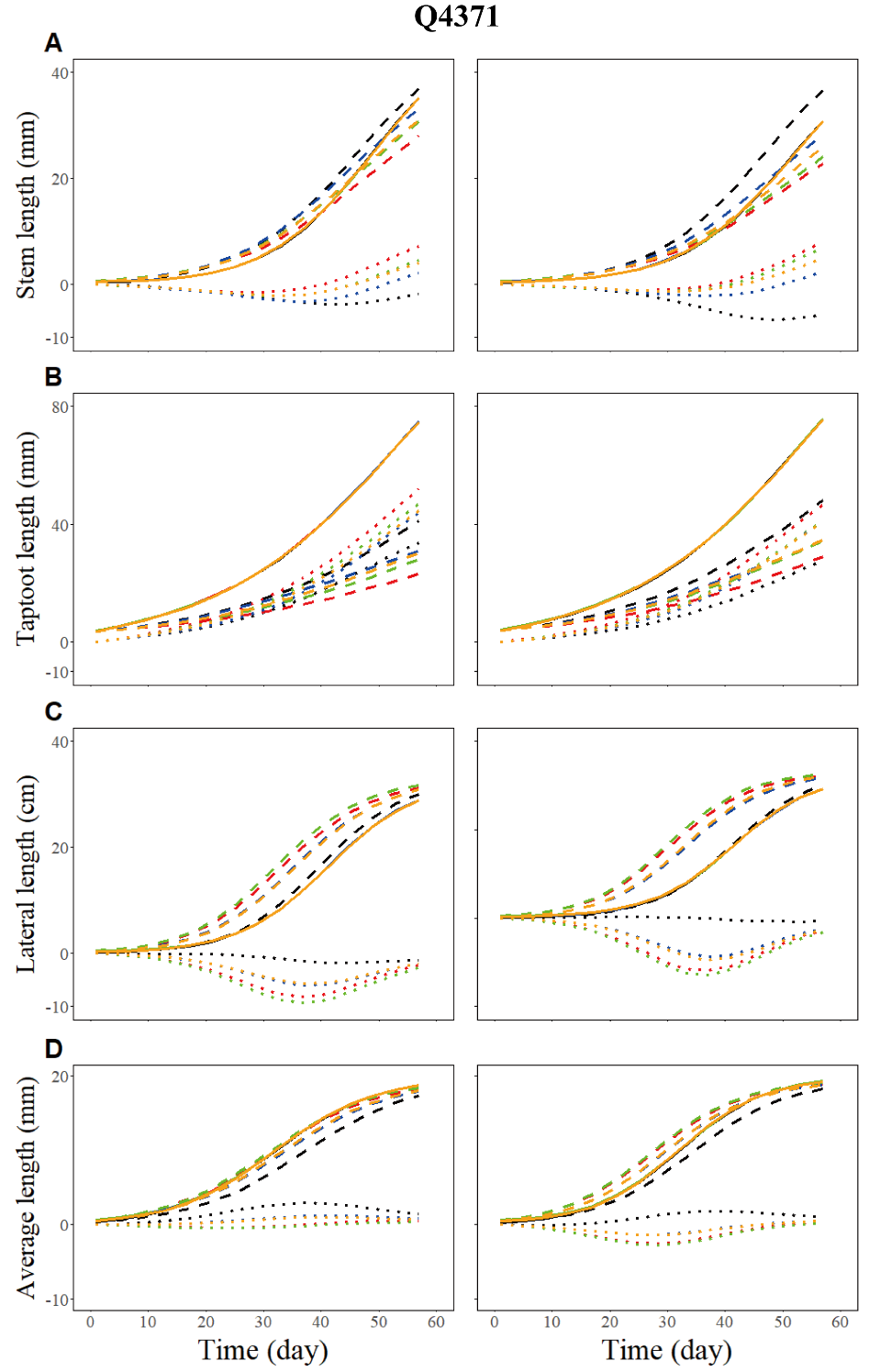


Fig. S9. Simulation results of heritability 0.05 (red lines) and 0.1 (green lines) when the sample size is 100, and simulation results of the heritability 0.05 (blue lines) and 0.1 (orange lines) when the sample size is 345 (Q4371). Simulated growth curves (dotted lines) of heterozygote and homozygote for stem length (A), taproot length (B), lateral root length (C) and the average lateral root length (D) are compared with the real curves (black lines). The solid lines represent the overall growth curves, the broke lines represent the independent growth curves, and the dot lines represent one trait is influenced by the other three traits.


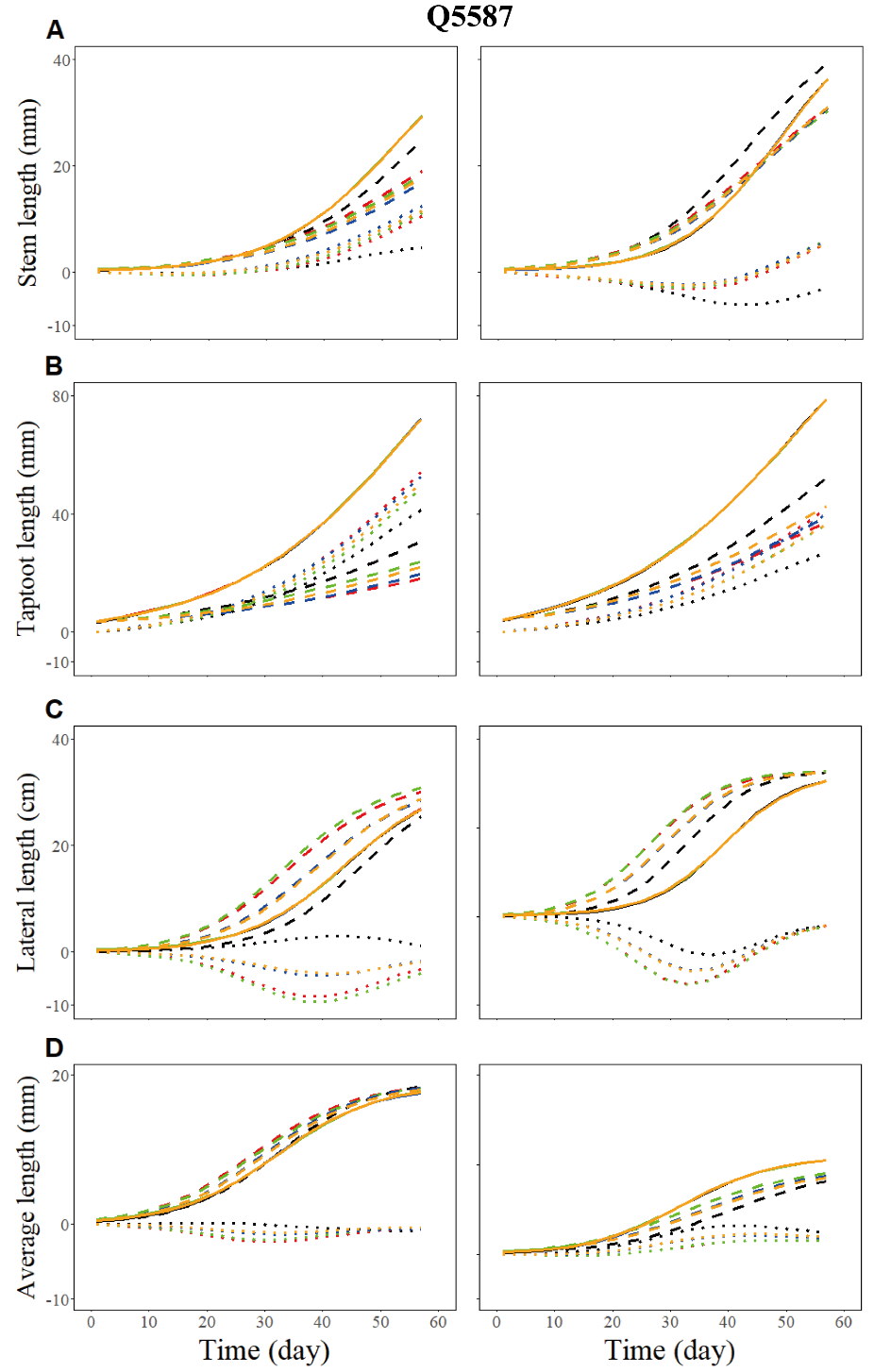


Fig. S10. Simulation results of heritability 0.05 (red lines) and 0.1 (green lines) when the sample size is 100, and simulation results of the heritability 0.05 (blue lines) and 0.1 (orange lines) when the sample size is 345 (Q5587). Simulated growth curves (dotted lines) of heterozygote and homozygote for stem length (A), taproot length (B), lateral root length (C) and the average lateral root length (D) are compared with the real curves (black lines). The solid lines represent the overall growth curves, the broke lines represent the independent growth curves, and the dot lines represent one trait is influenced by the other three traits.


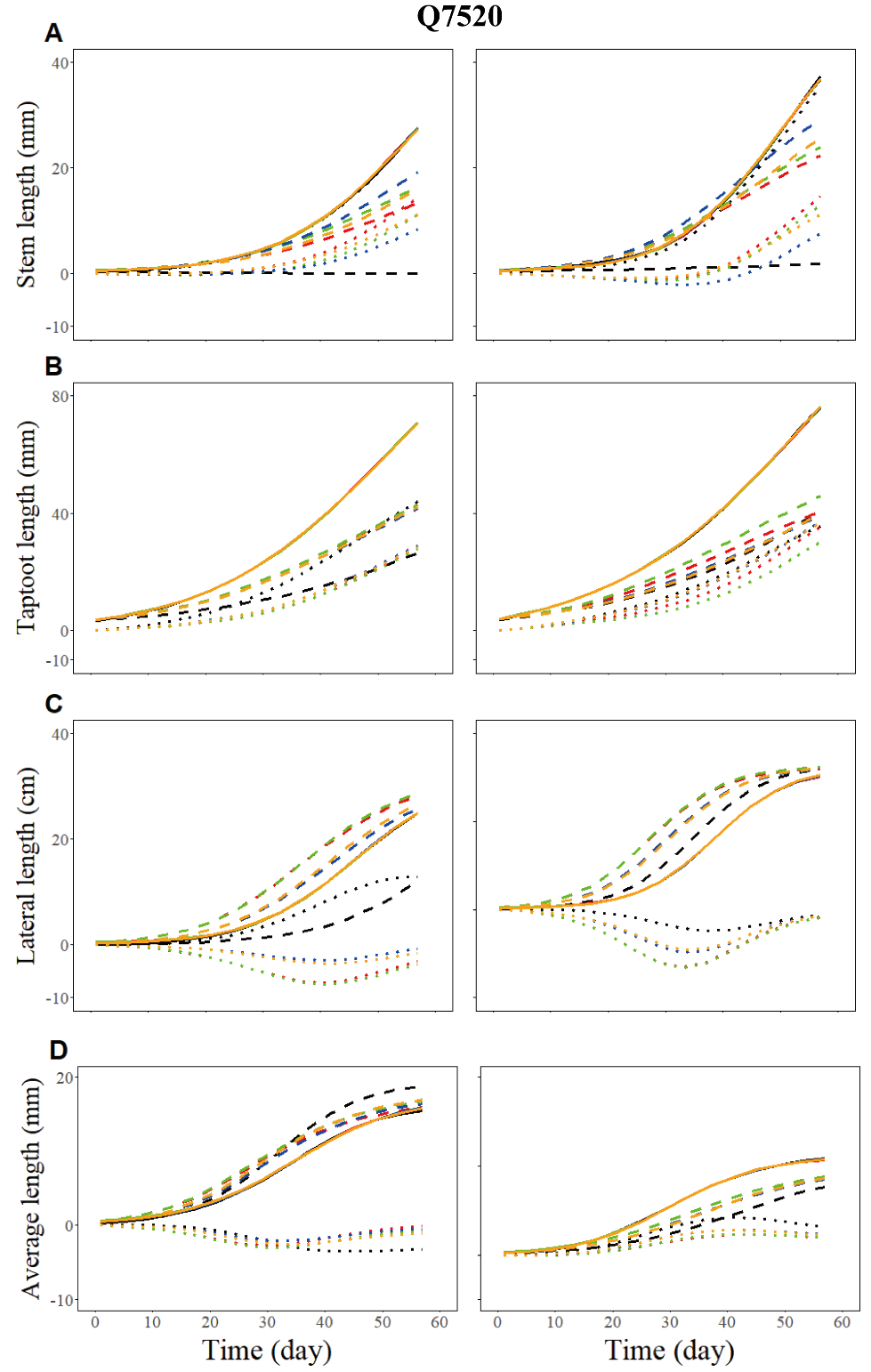


Fig. S11. Simulation results of heritability 0.05 (red lines) and 0.1 (green lines) when the sample size is 100, and simulation results of the heritability 0.05 (blue lines) and 0.1 (orange lines) when the sample size is 345 (Q7520). Simulated growth curves (dotted lines) of heterozygote and homozygote for stem length (A), taproot length (B), lateral root length (C) and the average lateral root length (D) are compared with the real curves (black lines). The solid lines represent the overall growth curves, the broke lines represent the independent growth curves, and the dot lines represent one trait is influenced by the other three traits.
